# Fluid Osmolarity Modulates the Rate of Spontaneous Contraction of Lymphatic Vessels and Lymph Flow by Means of a Cooperation between TRPV and VRAC Channels

**DOI:** 10.3390/biology12071039

**Published:** 2023-07-23

**Authors:** Eleonora Solari, Cristiana Marcozzi, Daniela Negrini, Andrea Moriondo

**Affiliations:** Department of Medicine and Technological Innovation (DIMIT), Università degli Studi dell’Insubria, 21100 Varese, Italy; eleonora.solari@uninsubria.it (E.S.); cristiana.marcozzi@uninsubria.it (C.M.); daniela.negrini@uninsubria.it (D.N.)

**Keywords:** TRPV, VRAC, lymphatic vessel, physiology, vessel contractility, lymph flow, osmolarity

## Abstract

**Simple Summary:**

The ability of lymphatic vessels to achieve lymph transport by exploiting external forces acting upon the vessels or by an intrinsic pumping mechanism based on spontaneous contractions of the vessel wall is vital for the physiological drainage of interstitial fluid. The osmolarity of the microenvironment can alter the spontaneous contraction rate and therefore modify lymph formation and propulsion. Several ion channels can be involved in the molecular mechanisms underlying this response; notably, transient receptor potential channels of type V4 and V1 and volume-regulated anion channels are among them and are expressed by lymphatic vessels. In this work, we provide functional evidence that the response to hyperosmolarity and hypo-osmolarity are mediated by these three channels in a cooperative way. TRPV4 and TRPV1 channels mostly mediate both the long-term responses, while VRACs are responsible for the early increase in the contraction rate characteristic of the exposure of vessels to a hyposmolar environment.

**Abstract:**

Lymphatic vessels are capable of sustaining lymph formation and propulsion via an intrinsic mechanism based on the spontaneous contraction of the lymphatic muscle in the wall of lymphatic collectors. Exposure to a hyper- or hypo-osmolar environment can deeply affect the intrinsic contraction rate and therefore alter lymph flow. In this work, we aimed at defining the putative receptors underlying such a response. Functional experiments were conducted in ex vivo rat diaphragmatic specimens containing spontaneously contracting lymphatic vessels that were exposed to either hyper- or hypo-osmolar solutions. Lymphatics were challenged with blockers to TRPV4, TRPV1, and VRAC channels, known to respond to changes in osmolarity and/or cell swelling and expressed by lymphatic vessels. Results show that the normal response to a hyperosmolar environment is a steady decrease in the contraction rate and lymph flow and can be prevented by blocking TRPV1 channels with capsazepine. The response to a hyposmolar environment consists of an early phase of an increase in the contraction rate, followed by a decrease. The early phase is abolished by blocking VRACs with DCPIB, while blocking TRPV4 mainly resulted in a delay of the early response. Overall, our data suggest that the cooperation of the three channels can shape the response of lymphatic vessels in terms of contraction frequency and lymph flow, with a prominent role of TRPV1 and VRACs.

## 1. Introduction

The lymphatic system drains fluid, solutes, and cells from the surrounding interstitial tissues through lymphatic capillaries, thereby enabling the formation of lymph. Lymph is also collected from pleural and peritoneal cavities, contributing to tissue fluid homeostasis and ensuring the proper coupling between the lungs and chest wall. Lymph is then propelled through larger collecting vessels along the lymphatic network and lymph nodes, and it is returned to the venous blood system [1,2,3].

Lymph formation and propulsion rely on hydraulic pressure gradients between the interstitium and the vessel lumen, as well as across successive lymphangions, which are the functional units [4] of the lymphatic system. These mechanisms depend upon two distinct processes known as extrinsic and intrinsic pumping [3,5]. The extrinsic mechanism involves mechanical stresses exerted by adjacent tissues, such as the contraction of skeletal or cardiac muscle fibers [6,7,8], cardiogenic activity [9], respiratory movements [10], and arterial vasomotion [11]. On the other hand, the intrinsic mechanism is reliant on the spontaneous contractions of lymphatic muscle (LM) that form a dense mesh within the vessel wall [12,13,14,15,16]. The intrinsic contractility of LM is driven by the pacemaker activity of lymphatic muscle cells, resulting from chloride-dependent STDs (spontaneous transient depolarizations) [17,18,19] and/or I_f_-like currents mediated by HCN (hyperpolarization-activated cyclic nucleotide-gated) channels [20,21,22]. This intrinsic pumping mechanism plays a pivotal role in maintaining lymphatic function in body districts where significant tissue displacement is absent, and it can be modulated by various drugs [23].

In pleural and diaphragmatic lymphatics, both extrinsic and intrinsic mechanisms cooperate to ensure proper lymph flow, with the contractions of the skeletal muscle serving as the primary force affecting lymphatic function [24,25,26]. However, lymphatics located at the periphery of the diaphragmatic dome, where mechanical forces are less pronounced and unevenly distributed, exhibit spontaneous contractions and possess a well-organized LM layer within the vessel wall [27,28]. The intrinsic contractility of diaphragmatic lymphatics is strongly influenced by the properties of the surrounding microenvironment, including local tissue temperature, which is sensed via TRPV4 (transient receptor potential cation channels, subfamily V, member 4) channels. Notably, an increase in temperature leads to an increment in lymph flow [29,30]. Fluid osmolarity in the interstitial space also significantly affects intrinsic contractility and lymph flow. Indeed, a hypertonic environment markedly reduces the intrinsic contraction frequency (f_c_), whereas a hypotonic environment transiently increases f_c_ [31].

In the last two decades, considerable attention has been devoted to studying the molecular mechanisms underlying the sensing of different stimuli, particularly involving TRP (transient receptor potential) channels, which are widely distributed throughout various body districts, including vascular beds [32,33]. These channels are known to be activated by multiple stimuli, including mechano-related processes such as osmosensing and volume sensing [34]. In particular, TRPV1 channels (transient receptor potential cation channels, subfamily V, member 1) are typically activated by noxious heat, low pH, capsaicin, and hypertonic conditions [35,36,37]. Conversely, TRPV4 channels respond to moderate heat, cell swelling, and shear stress [38,39,40].

In this study, we investigated the potential involvement of TRPV1 and TRPV4 channels in the molecular mechanisms underlying osmotic stress sensing in rat diaphragmatic lymphatics, as we previously demonstrated their presence in these vessels. To assess their role in sensing microenvironmental osmolarity, we used nonselective (Ruthenium Red) and selective TRPV channel blockers (capsazepine and HC-067047 for TRPV1 and TRPV4, respectively), in combination with hypertonic (324 mOsm) or hypotonic (290 mOsm) solutions.

Furthermore, using the selective blocker DCPIB, we also explored the possible contribution of VRACs (volume-regulated anion channels) [41] to the response to hyposmolarity, as osmotic cell swelling increases their opening probability. Our data suggest that TRPV1 channels are deeply involved in the molecular mechanism underlying the sensing of a hypertonic microenvironment in rat diaphragmatic lymphatics, while VRACs, rather than TRPV4 channels, play the primary role in sensing and responding to hypotonic conditions.

## 2. Materials and Methods

### 2.1. Surgical Procedures and In Vivo Lymphatics Staining

All of the experiments performed in this study were conducted in accordance with the guidelines of the University of Insubria Animal Care and Use Ethics Committee (OpBA) and approved by the Italian Ministry of Health, according to D.Lgs 26/2014 (experimental protocol 698/2020-PR). The experiments involved 39 adult Wistar rats of both sexes (body weight, BW, 458 ± 23 g) that were raised in our in-house colony. The rats were housed in standard transparent plastic cages (Tecniplast SpA; Buguggiate, Varese, Italy) in groups of two to three littermates, with ad libitum access to food and water. To promote environmental enrichment, transparent plastic tunnels were provided as hiding places, and animals were allowed a 12 h:12 h light–dark cycle.

Deep anaesthesia was induced using an anaesthetic cocktail of 75 mg/Kg BW Ketamine (Lobotor, ACME S.r.l., Corte Tegge (RE), Italy) and 0.5 mg/Kg BW Medetomidine (Domitor, Vétoquinol Italia S.r.l., Bertinoro (FC), Italy) in saline solution, administered intraperitoneally. An additional half bolus was administered after 60 min, while continuously checking the appropriate level of anesthesia by observing the absence of the noxious plantar reflex in the hind paw.

In vivo staining of diaphragmatic lymphatic vessels was performed following a well-established protocol [25,28] using fluorescent-conjugated high-molecular-weight dextrans, which can be drained by lymphatic vessels in serosal cavities due to the nonselectivity of lymphatic capillaries but cannot enter blood vessels due to their large size. Briefly, a 0.8 mL bolus of 2% FITC-conjugated dextrans (250 kDa, Ex/Em: 505/515; FD250S, Merck, Milan, Italy) in saline was administered intraperitoneally to deeply anesthetized animals using a stainless-steel injecting cannula (~0.8 mm outer diameter). The cannula was inserted through the abdomen and carefully positioned in the subdiaphragmatic region. Subsequently, the animals were placed in the prone position on a warmed (37 °C) blanket and left to spontaneously breathe, allowing the fluorescent dye to be drained by the diaphragmatic lymphatic network. After 60 min, the animals underwent tracheotomy, and a T-shaped cannula was inserted into the trachea. The rats were then mechanically ventilated (Inspira; Harvard Apparatus, Holliston Massachusetts, USA) with room air at tidal volume and the respiratory rate automatically set by the instrument based on the animals’ body weight. The chest wall was opened to expose the pleural diaphragmatic surface, and FITC-filled lymphatic vessels were visualized under a stereomicroscope (SV11 fitted with a 1× frontal lens, Zeiss, Milan, Italy) equipped with a custom-made LED fluorescence epi-illuminator (from Luxeonstar high-intensity LEDs; Luxeon Star Leds, Lethbridge, Alberta, CA). Throughout the procedure, great care was taken to prevent dehydration of the diaphragmatic tissue by repeatedly rinsing the tissue with a heated (37 °C) saline solution.

**Figure 1:**
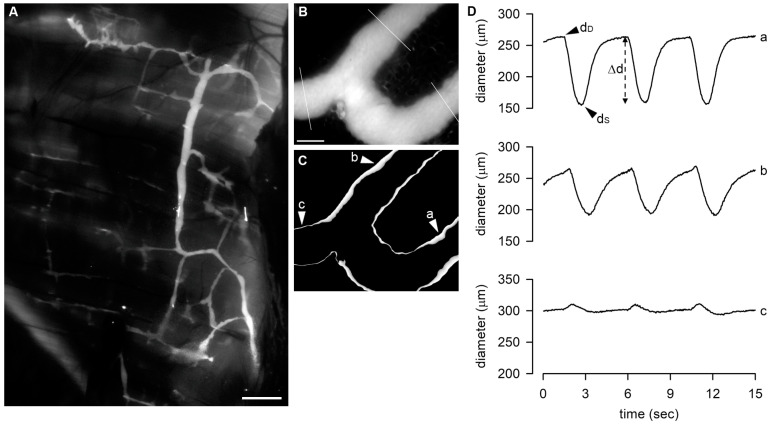
(**A**) Representative image of diaphragmatic lymphatic network, highlighted by an intraperitoneal injection of FITC-dextrans, showing the organization of lymphatic vessels on the pleural side of rat diaphragm. Scale bar is 1 mm. (**B**) Representative image of a FITC-filled lymphatic vessel taken from the video recording of a spontaneously contracting tract with the indication of the 3 sites in which the diameter was measured. Scale bar is 200 μm. (**C**) STD analysis of the vessel wall of the same lymphatic tract shown in (**B**), emphasizing contracting sites (a and b), characterized by the thicker white edges highlighting the vessel wall motion, and the not-contracting one (c). (**D**) Plot of diameter profiles over time measured in a, b, and c (as indicated in panels (**A**,**B**)). Sites a and b display the typical diastolic-to-systolic contracting cycle. d_D_, end-diastolic diameter; d_S_, systolic diameter; Δd, contraction amplitude.

Lymphatic vessels displaying spontaneous contractions were typically identified at the far muscular periphery. Suitable contracting lymphatics were video-recorded in vivo for 5 min at 10 fps by using a charge-coupled device (CCD) camera (Orca ER; Hamamatsu, Milan, Italy) connected to a PC running the proprietary SimplePCI Software (Hamamatsu). In Figure 1A, we report a still image of a large part of a FITC-stained lymphatic network on the pleural side of the diaphragm in vivo and in situ before the explant of tissue samples. Up to 4–5 diaphragmatic specimens containing contracting lymphatics were carefully excised from each animal, ensuring the preservation of the proper geometry of the vessel network and the functionality of both lymphatic vessels and surrounding tissue [25,42]. Diaphragmatic tissue strips (up to 35–40 mm in length and 5–10 mm in width) were excised from the costal margin to the central tendon and placed in petri dishes filled with cold HEPES-buffered Tyrode’s solution (containing, in mM: 119 NaCl, S7653; 5 KCl, P9541; 25 HEPES buffer, H3375; 2 CaCl_2_, 21115; 2 MgCl_2_, 63069; 33 D-glucose, G5767; pH = 7.4; all products were purchased from Merck; Cold Spring Harbor Protocols, doi:10.1101/pdb.rec10805) at 308 mOsm (*storage* solution). The tissue was kept at 4 °C in the dark until it was used for ex vivo analysis.

At the end of the surgical procedure involving the collection of 4–5 diaphragmatic tissue strips from one diaphragm, tissue samples collected in Petri dishes in HEPES-Tyrode solution were stored at 4 °C until use in the subsequent ex vivo experiments, and animals were euthanized by an overdose of the anaesthetic cocktail.

### 2.2. Ex Vivo Experimental Setup

Each diaphragmatic tissue strip was pinned to the bottom of a custom-made 3D-printed perfusion chamber with careful attention to maintain the geometry and dimensions of the lymphatic network as observed in situ to prevent any mechanical stress-related artifacts on spontaneous contractility. The perfusion chamber was filled with oxygenated Hepes-buffered Tyrode’s solution and placed on the stage of an upright microscope (BX51WI, Olympus, Milan, Italy). Lymphatics were visualized in epifluorescence with a dry 4× Olympus Plan APO objective (numerical aperture = 0.13). A black and white camera (WAT-902H, Watec, from Sicom snc, Como, Italy) connected to a PC running VirtualDub software (http://www.virtualdub.org, accessed on 29 May 2023) was used to video-record contracting lymphatics at a frame rate of 25 fps. A sample image of a small branching of a lymphatic collector acquired through the Olympus BX51WI is presented in Figure 1B. On the image, there is the superimposition of the three sites where diameter measurements were made offline from the acquired video recording.

As tissue temperature has a profound impact on the spontaneous contractility of lymphatic vessels [29], the temperature of the bathing solution was continuously checked and recorded by means of an implantable T thermocouple placed as close as possible to the contracting vessel. The bath temperature was maintained at 35.0 ± 0.1 °C using the ITC100-VH PID thermostat (Inkbird, Shenzhen, China), which controlled a resistive load embodied into the recording chamber. The careful repositioning of the diaphragmatic tissue strip inside the recording chamber and the temperature control assured that experiments were performed in conditions very close to the in vivo one.

### 2.3. Solutions Used

The solutions used at various osmolarities are summarized in Table 1. All solutions were prepared based on the previously described HEPES-buffered Tyrode’s solution. D-mannitol (M4125, Merck), a widely used non-metabolically active molecule known to have negligible effects on lymphatic intrinsic contractility at 37 °C [31], was used to half replace the D-glucose in the *storage* solution to obtain the isosmotic *control*_308_ solution. Hypertonic and hypotonic reference solutions (*hyper*_324_ and *hypo*_290_, respectively) were obtained by adjusting the amount of D-mannitol. The actual osmolarity of all solutions used in the ex vivo experiments was measured using a micro-osmometer (Hermann Roebling, Messtechnik, Berlin, Germany). The hypo- to hyperosmotic range selection was based on previous data [31], which demonstrated reproducible results within the normal range of rat plasma osmolarity (288–336 mOsm) [43]. The different drugs used in the experiments were diluted in the aforementioned bathing solutions (Table 1).

### 2.4. Stability Test

To assess the impact of D-mannitol half substitution for D-glucose on lymphatic intrinsic contractility at a temperature of 35 °C, comparative experiments were performed. A time-course test was performed to measure the intrinsic contraction frequency (f_c_), wherein lymphatics (*n* = 5) were video-recorded for 5 min in the *storage* solution. Subsequently, the bath solution was replaced with the isosmotic *control*_308_ (Table 1A), and lymphatics were further recorded for an additional 25 min. Intrinsic f_c_ data were expressed as a percentage of the f_c_ displayed in the *storage* solution (set as 100% at t_0_) and compared to previous data recorded at 37 °C [31].

### 2.5. Impact of Fluid Osmolarity on Intrinsic Contraction Frequency at 35 °C

Reference curves for the effects of osmolarity on intrinsic f_c_ at 35 °C were obtained for hypertonic (324 mOsm) and hypotonic (290 mOsm) conditions (*hyper*_324_ and *hypo*_290_ groups in Table 1, respectively). The experiments were carried out by raising the initial temperature (~20 °C) of the perfusion chamber to a stable value of 35.0 ± 0.1 °C in the *storage* solution. Tissue samples containing contracting lymphatics were then bathed in the *control*_308_ solution, and video recordings were made for 5 min (baseline condition). Subsequently, the perfusing solution was switched to either *hyper*_324_ or *hypo*_290_, and the vessels’ contractility was recorded for an additional 20 min. The recording time did not exceed 25 min as pointed out by the stability test to avoid possible data inconsistencies due to prolonged exposure to D-mannitol-based solutions.

### 2.6. Functional Analysis

To investigate the osmosensing mechanisms involved in the response to changes in surrounding fluid osmolarity, four sets of ex vivo functional experiments were randomly conducted to evaluate the effects of a hypertonic environment on the spontaneous lymphatic contractility, whereas five were performed for the effects of a hypotonic one.

In the RuR experimental groups (refer to Table 1B for hypertonic solutions and Table 1C for hypotonic solutions), lymphatic vessels were acclimated at 35 °C in the isosmotic *control*_308_ solution for 3 min. Lymphatics were then challenged with the nonselective TRPV1–6 channel antagonist Ruthenium Red (RuR, 557450, Merck, at a concentration of 10–20 µM) in *control*_308_ for 5 min. Subsequently, the perfusing solution was switched to either *hyper*_324_ or *hypo*_290_ in which RuR was diluted to a final concentration of 10 or 20 µM (RuR_10_ and RuR_20_ groups, respectively), and the vessels were video-recorded for an additional 15 min.

In the HC experimental groups, the same procedure as the RuR groups was followed. Contracting lymphatics were recorded in the baseline condition (isosmotic *control*_308_) for 3 min, and then they were exposed to the selective TRPV4 channel blocker HC-067047 (HC, SML0143, Merck; at a concentration of 2.5–10 µM) in *control*_308_ for 5 min. Subsequently, the bathing solution was switched to *hyper*_324_ or *hypo*_290_ solution containing HC at a final concentration of 2.5–10 µM (HC_2.5_, HC_5_, and HC_10_ groups in Table 1B,C), and the contractile activity was recorded for 15 min.

For the caps experimental groups, contracting vessels were recorded for 3 min in the baseline condition (isosmotic *control*_308_) and then challenged with the selective TRPV1 channel blocker capsazepine (caps, 0464, Tocris Bioscience; at a concentration of 5–10 µM) according to the same experimental procedure of the RuR and HC groups (caps_5_ and caps_10_ groups in Table 1B,C).

Ruthenium Red was prepared as a stock solution (10 mM) dissolved in water, whereas HC-067047 (10 mM), capsazepine (25 mM), and DCPIB (25 mM) were dissolved in DMSO (dimethyl sulfoxide; 41639; Merck) to prepare stock solutions. Aliquots were stored at −20 °C until diluted in the appropriate bathing solution to achieve the final concentration.

To test for possible vehicle-induced effects, if any, *DMSO*_0.1%_ experimental groups were included (refer to Table 1B,C), where DMSO was dissolved at a final concentration of 0.1% (maximum DMSO concentration in the bathing solutions). The same protocol was performed for the *DMSO*_0.1%_ groups. Lymphatic spontaneous activity was video-recorded for 3 min in *control*_308_, then for 5 min in *control*_308_ plus 0.1% DMSO, and, finally, the bathing solution was switched to either a 324 mOsm or 290 mOsm solution containing 0.1% DMSO.

In the DCPIB experimental group, lymphatic spontaneous contractile activity was recorded in the baseline condition (isosmotic *control*_308_) for 3 min. Subsequently, the vessels were exposed to the volume-regulated anion channel (VRAC) blocker DCPIB (1540, Tocris Bioscience, Milan, Italy) at a final concentration of 5 µM in *control*_308_ for 5 min. Lymphatics were then bathed with 5 µM DCPIB diluted in the 290 mOsm hypotonic buffer (DCPIB_5_ group) for 15 min. DCPIB was specifically applied only to the hypotonic environment experimental group (refer to Table 1C).

In order to minimize possible artifacts due to tissue temperature oscillations, repeated tests, and/or drug cross-reaction, each set of experiments (*control*_308_, *hyper*_324_, *hypo*_290_, RuR, HC, caps, *DMSO*_0.1%_, and DCPIB) followed the guidelines mentioned below: (a) maintain a stable temperature of 35.0 ± 0.1 °C; (b) video-record only one spontaneous contracting vessel for each diaphragmatic tissue strip; (c) consider only one osmotic and/or drug condition (as summarized in Table 1) at a time; and (d) use a 3D-printed custom-made disposable perfusion chamber.

### 2.7. Real-Time PCR Assay for VRACs

Four animals were used to obtain diaphragmatic tissue specimens containing lymphatic networks, which were then pinned to the bottom of the perfusion chamber. The chamber was filled with the *storage* solution and placed on a custom-made 3D-printed support under the SV11 stereomicroscope. Lymphatic vessels were video-recorded ex vivo for 1 min using the Hamamatsu CCD camera while the tissue temperature in the perfusion chamber was continuously monitored and maintained at 35.0 ± 0.1 °C.

The video data obtained were immediately exported, and contracting sites were identified using the “Standard Deviation Z-project Function” of ImageJ Software (National Institutes of Health; https://imagej.nih.gov/ij/, accessed on 29 May 2023) [44]. Indeed, contracting tracts exhibited the sharpest diameter change, highlighted by the software plugin as the thickest region of the vessel rims [45].

From each animal, two sets of samples were prepared: (a) spontaneously contracting lymphatics and (b) not-contracting vessels. These samples were carefully dissected from the surrounding interstitial tissue, immediately frozen in dry ice, and pooled separately to proceed with total RNA extraction (*n* = 4 samples each). Additionally, dorsal root ganglions (DRGs) were excised from the same animals and processed for total RNA extraction and real-time PCR assay as positive controls.

Total RNA extraction was performed using the Quick-RNA MicroPrep kit (R1050, Zymo Research, provided by Euroclone, Milan, Italy), and cDNA was obtained by using the HighCapacity cDNA Reverse Transcription Kit (4368814; ThermoFisher, Monza (MB), Italy) from 100 ng of total RNA. Real-time PCR was performed on an ABI Prism 7000 instrument (Applied Biosystems, Monza (MB), Italy) as previously reported [30]. The oligonucleotides for VRACs and β-actin were designed using CLC Main workbench software (Qiagen, Milan, Italy) and are reported in Table 2.

### 2.8. Data Analysis

Video data of FITC-fluorescent lymphatics obtained during ex vivo functional experiments were analysed offline. They were converted into black and white binary image sequences, and the Standard Deviation Z-project function of ImageJ Software was applied to short frame series that encompassed 4–5 complete intrinsic contraction cycles to identify intrinsically contracting sites. The binary video data were then analysed using the “Diameter” plugin [46] of ImageJ Software, which allows the visualization of diameter profiles over time, highlighting the end-diastolic diameter (d_D_, µm) and end-systolic diameter (d_S_, µm), which are characteristic of contracting lymphatics. In Figure 1B,C, we report the image of a FITC-filled lymphatic vessel tract as it appears in epifluorescence illumination onto the stage of a BX51WI microscope and the STD analysis performed on the video recorded from that particular site. The major contracting tracts along the vessel are represented by the sites indicated (a and b), where the apparent edge thickness in the processed image is larger compared to the rest of the vessel wall. Panel D shows the recordings of diameter length over time as obtained from the three sites indicated in panel B with the ImageJ plugin used.

The diameter traces were further examined using Clampfit 10 Software (Molecular Devices) to compute the lymphatic spontaneous contraction frequency (f_c_, cycles/min) over a time period of 1 min (or 30 s for the evaluation of the rapid early hypotonic-related response in the first 3–5 min of perfusion).

Moreover, the values of d_D_ and d_S_ for each contraction cycle were measured for all tested conditions. The contraction amplitude (Δd, µm) was computed as in Equation (1):

(1) $$∆d=d_{D}-d_{S}$$

and then it was expressed as a percentage of respective d_D_.

Since lymphatic vessels on the pleural side of the diaphragm are ellipse-shaped, with a transverse to parallel diameter ratio of 0.35:1 [28,47], the diastolic-to-systolic reduction in cross-sectional area (ΔS, µm^2^) was computed as in Equation (2):

(2) $$∆S=\left[\left(r_{D}\cdot \left(r_{D}\cdot 0.35\right)\right)\cdot \pi \right]-\left[\left(r_{S}\cdot \left(r_{S}\cdot 0.35\right)\right)\cdot \pi \right]$$

with r_D_ and r_S_ being mean end-diastolic and end-systolic radii (µm), respectively.

The volume of lymph ejected during each single contraction was computed for a vessel tract having a length of 105.50 µm, which had been previously identified as the ideal lymphatic tract [29]. As lymph flow rate (*J_lymph_*, nL/min) in diaphragmatic vessels is laminar [25], *J_lymph_* was estimated according to (Equation (3)):

(3) $$J_{lymph}=\frac{\left(∆S\cdot 105.5\;\mu m\right)\cdot f_{c}}{10^{6}}$$

with 10^6^ being the µm^3^ to nl conversion factor. Data were then expressed as a percentage of *J_lymph_* value at t_0_.

Data fitting was performed using four-parameter logistic curves (Equation (4)):

(4) $$Y=bottom+\frac{\left(top-bottom\right)}{1+{\left(\frac{X}{X_{50}}\right)}^{Hillslope}}$$

where the bottom and top represent the Y values at the lower and higher plateaus, respectively. The inflection point value, X_50_, indicated the time interval corresponding to the half-maximal effect on spontaneous contractility and was referred to as f_c50_ or J_50_ depending on whether Y represented intrinsic f_c_ or *J_lymph_*, respectively. The Hillslope parameter defined the steepness of the curve.

### 2.9. Statistical Analysis

All data are presented as mean ± standard error (SE). Statistical analysis and data fitting were performed using SigmaPlot 10.0 (Systat Software, San Jose, CA, USA) and GraphPad Prism 5 software. Significance between means was assessed using one-way ANOVA with Bonferroni post-test for multiple comparisons and paired or unpaired Student’s *t*-test after a data normality distribution check. The threshold for statistical significance was set at *p* < 0.05.

## 3. Results

### 3.1. Stability Test, Effect of Changes in Fluid Osmolarity on Intrinsic f_c_ at 35 °C, and Preliminary Test of Channels Blockers

**Figure 2:**
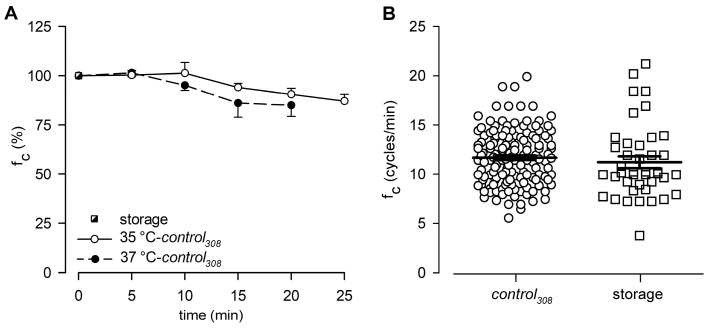
(**A**) Time course of mean lymphatic intrinsic f_c_ during *control*_308_ perfusion recorded either at 35 °C (hollow circles and solid line, *n* = 5) or 37 °C (filled circles and dashed line, *n* = 5), expressed as % of f_c_ displayed at t_0_ in *storage* solution at the very same bathing temperature (black and white square). At 20 min, f_c_ was not statistically different (*p* = 0.748, Student’s *t*-test, *n* = 10). (**B**) Plot of intrinsic f_c_ displayed by diaphragmatic contracting lymphatics either perfused with *control*_308_ (hollow circles, *n* = 141) or *storage* (hollow squares, *n* = 41) solutions at 35 °C.

At the beginning of stability tests, lymphatic vessels bathed with the *storage* solution at 35 °C displayed an intrinsic f_c_ of 10.9 ± 0.9 cycles/min, which was set as the reference value at t_0_ (100%, Figure 2A). Then, intrinsic f_c_ slightly decreased over time when vessels were challenged with the D-mannitol-based *control*_308_ solution at 35 °C, reaching a value of about 90.5 ± 3.0% of that at t_0_ after 20 min (9.9 ± 0.8 cycles/min, Figure 2A). At 25 min, intrinsic f_c_ was 87.1 ± 3.5% of the t_0_ value (9.5 ± 0.7 cycles/min), as the one obtained in a previous work [31] when vessels were bathed with a *control*_308_ solution for 20 min at 37 °C (84.9 ± 5.6% of f_c_ at t_0_; Figure 2A). Based on this result, we confirmed that even at the lower temperature of 35 °C, the half replacement of D-glucose with the non-polar agent D-mannitol did not significantly affect lymphatic intrinsic f_c_.

In this work, a total of 141 spontaneously contracting lymphatics were tested, displaying an overall mean f_c_ of 11.7 ± 0.2 cycles/min when perfused with the *control*_308_ solution at 35 °C (Figure 2B). The results were not significantly different from those previously recorded in diaphragmatic lymphatics bathed with a *storage* solution at the same 35 °C temperature (11.3 ± 0.6 cycles/min, *n* = 41, Figure 2B; data from experiments in Ref. [30], *p* = 0.3998, *control*_308_ vs. *storage*, Student’s *t*-test, *n* = 182).

Among the total 141 spontaneous contracting lymphatics, 61 vessels were randomly assigned to experimental groups to test the effect of the hypertonic environment (groups in Table 1B, mean f_c_ = 11.5 ± 0.3 cycles/min), whereas 80 were assigned to experimental groups to test the effect of the hypotonic environment (groups in Table 1C, mean f_c_ = 11.9 ± 0.3 cycles/min, *p* = 0.3530, hypo vs. hyper groups, Student’s *t*-test, *n* = 141).

**Figure 3:**
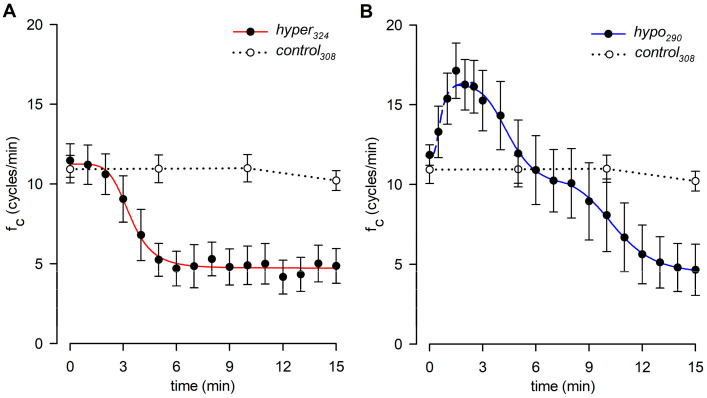
(**A**) Time course showing the effect of the hyperosmotic environment (*hyper*_324_, filled circles) on average lymphatic intrinsic f_c_. Data points were fitted by a four-parameter sigmoidal equation (red solid line). Time course of isosmotic *control*_308_ (hollow circles and dotted line) is reported for reference. (**B**) Time course showing the effect of the hyposmotic environment (*hypo*_290_, filled circles) on average lymphatic intrinsic f_c_. The rising period was fitted by a four-parameter sigmoidal equation (blue dashed line), whereas the following decreasing phases were fitted by two sigmoidal curves (blue solid line). Time course of isosmotic *control*_308_ (hollow circles and dotted line) is reported for reference.

Figure 3 shows the effect induced by changes in the surrounding fluid osmolarity on intrinsic lymphatic f_c_ at a stable temperature of 35 °C. Contracting lymphatics (*n* = 10, mean f_c_ in *control*_308_ = 11.5 ± 1.0 cycles/min at t_0_) bathed with the *hyper*_324_ solution displayed a time-dependent f_c_ decrease (Figure 3A), reaching a significantly lower value of 4.9 ± 1.1 cycles/min (*p* < 0.01 vs. t_0_, paired Student’s *t*-test, *n* = 10), which corresponded to a 60.1 ± 7.3% reduction from baseline f_c_. The plot in Figure 3A was fitted by a sigmoidal relationship (parameters for data fitting in supplemental Table S1). The average time interval to attain the lower steady state was 5.50 ± 0.64 min.

Different contracting vessels (*n* = 8, mean f_c_ in *control*_308_ = 11.8 ± 0.6 cycles/min at t_0_) challenged with *hypo*_290_ solution (Figure 3B) displayed a transient significant increase in f_c_, reaching a mean upper value of 18.4 ± 1.8 cycles/min (mean value of f_c_ peaks, *p* < 0.01 vs. t_0_, paired Student’s *t*-test, *n* = 8) corresponding to an increase of +53.9 ± 10.7% of f_c_ at t_0_. The mean time to peak was 1.88 ± 0.26 min, and the rising phase was fitted by a sigmoidal relationship (Figure 3B, parameters for data fitting are reported in supplemental Table S2A). After reaching the peak, f_c_ displayed a two-phase sigmoidal decrease (Figure 3B, parameters for data fitting are reported in supplemental Table S2B). The late phase attained a mean lower value of 4.7 ± 1.6 cycles/min, not significantly different from the *hyper*_324_ f_c_ endpoint (*p* = 0.9093, Student’s *t*-test, *n* = 18), corresponding to a f_c_ decrease of 62.4 ± 12.7%. However, the *hypo*_290_ response took a significantly longer time to attain the steady value (11.00 ± 0.68 min, *p* < 0.01 vs. *hyper*_324_, Student’s *t*-test, *n* = 18). Sigmoidal data fitting of the *hypo*_290_ response is reported in supplemental Table S2B.

**Figure 4:**
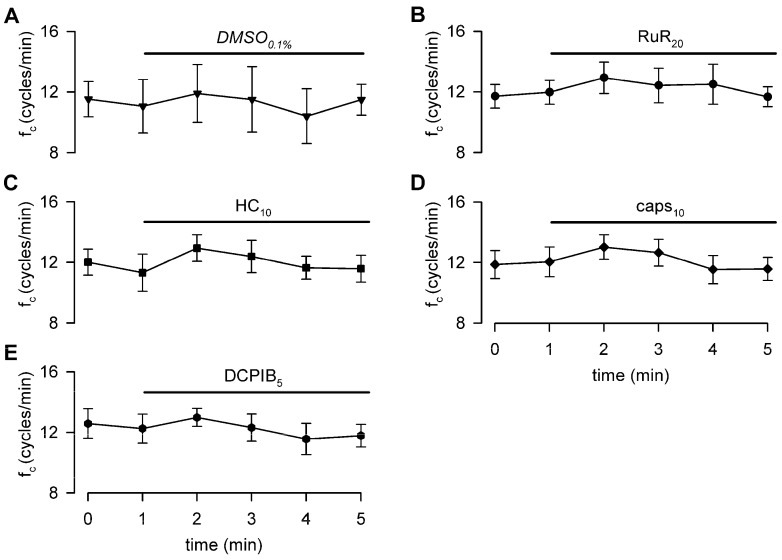
Time course of intrinsic f_c_ showing that neither the vehicle (panel (**A**)), nor the nonselective TRPV1-6 (**B**), nor the selective TRPV4 or TRPV1 (**C**,**D**) channel blockers affect lymphatic intrinsic contractility under isosmotic conditions. VRAC channel blocker (**E**) also does not affect lymphatic pacemaker activity when perfused under 308 mOsm conditions.

To rule out possible artifacts eventually induced by blockers of TRPV and VRAC channels on the lymphatic pacemaker, the intrinsic f_c_ was monitored during 5 min of perfusion under isosmotic conditions. No drug-related effects were found in any of the tested conditions on intrinsic contractility, as reported in Figure 4, which shows f_c_ data over time for the highest dose of each channel blocker used in functional experiments.

### 3.2. Effect of RuR, HC, and Capsazepine on Lymphatic Intrinsic Contractility in Hypertonic Solution

**Figure 5:**
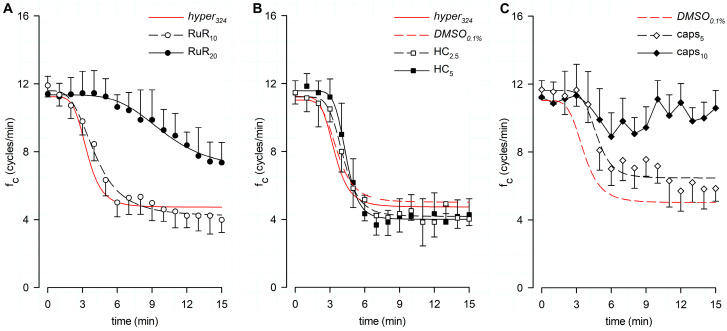
(**A**) Time course showing the effect of the nonselective TRPV1-6 channel blocker RuR in the 324-hypertonic solution on average f_c_ of spontaneous contracting lymphatics. Data from RuR_10_ (hollow circles) and RuR_20_ (filled circles) were fitted by four-parameter sigmoidal curves (black dashed line and black solid line, respectively). Time course of the effect of the hypertonic solution alone (*hyper*_324_) is reported for reference (red solid line). (**B**) Time course showing the effect of the TRPV4-selective blocker HC in the 324-hypertonic solution on average f_c_ of spontaneous contracting lymphatics. Data from HC_2.5_ (hollow squares) and HC_5_ (filled squares) were fitted by four-parameter sigmoidal curves (black dashed line and black solid line, respectively). Time courses of the effect of the hypertonic solution alone (*hyper*_324_, red solid line) and in presence of the vehicle (*DMSO*_0.1%_, red dashed line) are reported for reference. (**C**) Time course showing the effect of the TRPV1-selective blocker caps in the 324-hypertonic solution on average f_c_ of spontaneous contracting lymphatics. Data from caps_5_ (hollow diamonds) were fitted by a four-parameter sigmoidal curve (black dashed line). Exposure to caps_10_ (filled diamonds and black solid line) significantly reduced the response to the hypertonic environment. Time course of *DMSO*_0.1%_ (red dashed line) is reported for reference.

**Figure 6:**
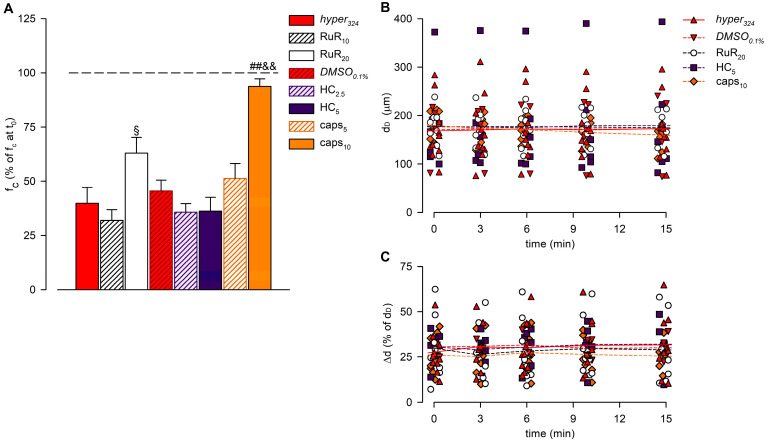
(**A**) Overall effect of different channel blockers in the 324-hypertonic solution on intrinsic f_c_, compared to *hyper*_324_ (red solid bar) or *DMSO*_0.1%_ (red striped bar), expressed as a percentage of f_c_ displayed at t_0_ (100%, identified by the black dashed line). The effects of the nonselective TRPV1-6 channel blocker Ruthenium Red (10 μM white striped bar, 20 μM white solid bar), as the selective TRPV4 channel blocker HC-067047 (2.5 μM purple striped bar, 5 μM purple solid bar) or the selective TRPV1 channel blocker capsazepine (5 μM orange striped bar, 10 μM orange solid bar) were tested. RuR_20_ significantly reduced the hyperosmolarity-induced decrease in intrinsic f_c_, and caps_10_ almost abolished the lymphatic f_c_ response to the hyperosmolar environment. All data are significantly lower than 100%, except caps_10_. ^§^ *p* < 0.05 vs. RuR_10_, ^##^ *p* < 0.01 vs. *DMSO*_0.1%_, ^&&^ *p* < 0.01 vs. caps_5_. (**B**) Lymphatic vessels’ end-diastolic diameter (d_D_) and (**C**) contraction amplitude (Δd, %) were not significantly affected by the application of different drugs (*hyper*_324,_ red up triangles and red solid line; *DMSO*_0.1%,_ red down triangles and red dashed line; RuR_20,_ hollow circles and black dashed line; HC_5,_ purple squares and purple dashed line; and caps_10,_ orange diamonds and orange dashed line).

Lymphatic vessels (*n* = 9, mean f_c_ at t_0_ = 11.9 ± 0.7 cycles/min) exposed to the nonselective TRPV1-6 channel blocker Ruthenium Red at a 10 μM dose (RuR_10_) displayed an intrinsic f_c_ time-dependent decrease (Figure 5A), attaining a lower value of 4.0 ± 0.8 cycles/min (*p* < 0.01 vs. t_0_, paired Student’s *t*-test, *n* = 9) after 15 min of perfusion. It corresponded to a 68.1 ± 4.9% reduction from the starting f_c_ (Figure 6A), similarly to data observed in *hyper*_324_. A different set of spontaneously contracting vessels (*n* = 8, mean f_c_ at t_0_ = 11.4 ± 1.0 cycles/min), exposed to a higher dose of RuR (20 μM, RuR_20_), displayed a less pronounced response to the hyperosmolar environment (Figure 5A), suggesting a TRPV involvement in hypertonic sensing. Indeed, after 15 min of perfusion with RuR_20,_ f_c_ attained a lower value of 7.4 ± 1.2 cycles/min (*p* < 0.01 vs. t_0_, paired Student’s *t*-test, *n* = 8) corresponding to a decrease of 37.1 ± 7.3% of the starting f_c_ (Figure 6A), significantly higher than RuR_10_ (*p* < 0.05, one-way ANOVA). Intrinsic f_c_ data for both RuR_10_ and RuR_20_ were fitted by sigmoidal curves (Figure 5A, parameters for data fitting are reported in supplemental Table S1).

A different set of lymphatics (*DMSO*_0.1%_, *n* = 4, mean f_c_ at t_0_ = 11.1 ± 0.6 cycles/min) was tested for possible vehicle-related artifacts on intrinsic f_c_, since the specific TRPV4 and TRPV1 channel blockers were dissolved in DMSO. After 15 min of *DMSO*_0.1%_ perfusion, intrinsic f_c_ significantly decreased, attaining a lower value of 4.9 ± 0.3 cycles/min (*p* < 0.01 vs. t_0_, paired Student’s *t*-test, *n* = 4; corresponding to a reduction of 54.5 ± 5.0% of the starting f_c_, Figure 6A), not significantly different from *hyper*_324_. Data fitting was performed by using a four-parameter sigmoidal curve (Figure 5B, whose parameters are reported in supplemental Table S1; data points not shown). When lymphatics (*n* = 6, mean f_c_ at t_0_ = 11.5 ± 0.7 cycles/min) were challenged with the selective TRPV4 channel blocker HC-067047 at a 2.5 μM dose (HC_2.5_), no involvement was found in the response to hyperosmolarity, as f_c_ decreased similarly to DMSO_0.1%_. Intrinsic f_c_ attained a comparable steady value of 4.0 ± 0.4 cycles/min after 15 min (*p* < 0.01 vs. t_0_, paired Student’s *t*-test, *n* = 6), corresponding to a reduction of 64.3 ± 4.0% of the starting f_c_ (Figure 5B and Figure 6A). Moreover, when a different set of lymphatics (*n* = 6, mean f_c_ at t_0_ = 11.5 ± 0.7 cycles/min) was exposed to 5 μM HC (HC_5_), intrinsic f_c_ significantly decreased, attaining a lower value of 4.3 ± 0.9 cycles/min (*p* < 0.01 vs. t_0_, paired student’s *t*-test, *n* = 6), corresponding to a decrease of 63.8 ± 6.5% of the starting f_c_ (Figure 5B and Figure 6A). As a result, no significant differences were found between *DMSO*_0.1%_ and both HC groups (*p* = 0.4403, one-way ANOVA). Data sets for both HC_2.5_ and HC_5_ groups were fitted by four-parameter sigmoidal curves (Figure 5B, parameters for data fitting are reported in supplemental Table S1). A different set of lymphatics (*n* = 10, mean f_c_ at t_0_ = 11.7 ± 0.5 cycles/min) was challenged with the selective TRPV1 channel blocker capsazepine at a final concentration of 5 µM (caps_5_), displaying a less pronounced f_c_ decrease in response to hyperosmolarity. Intrinsic f_c_ was found to be 5.9 ± 0.8 cycles/min (*p* < 0.01 vs. t_0_, paired Student’s *t*-test, *n* = 10), corresponding to a reduction of 48.7 ± 6.9% of the starting f_c_ after 15 min of perfusion (Figure 5C and 6A). Data from caps_5_ experiments were fitted by a four-parameter sigmoidal curve (Figure 5C, parameters for data fitting are reported in supplemental Table S1). When lymphatics (*n* = 8, mean f_c_ at t_0_ = 11.2 ± 1.0 cycles/min) were exposed to 10 μM capsazepine (caps_10_, Figure 5C), the spontaneous contractility remained quite unaffected, being f_c_ 10.6 ± 1.0 cycles/min after 15 min (−6.3 ± 3.5% of f_c_ at t_0_, Figure 6A), a value almost similar to the starting f_c_ and significantly higher than both *DMSO*_0.1%_ and caps_5_ (*p* < 0.01 one-way ANOVA).

The time-related effect on intrinsic f_c_ induced by the hypertonic environment alone (*hyper*_324_) or in the presence of any other channel blocker tested (expressed as a percentage of f_c_ displayed at t_0_) and the statistical analysis are summarized in Figure 6A.

**Figure 7:**
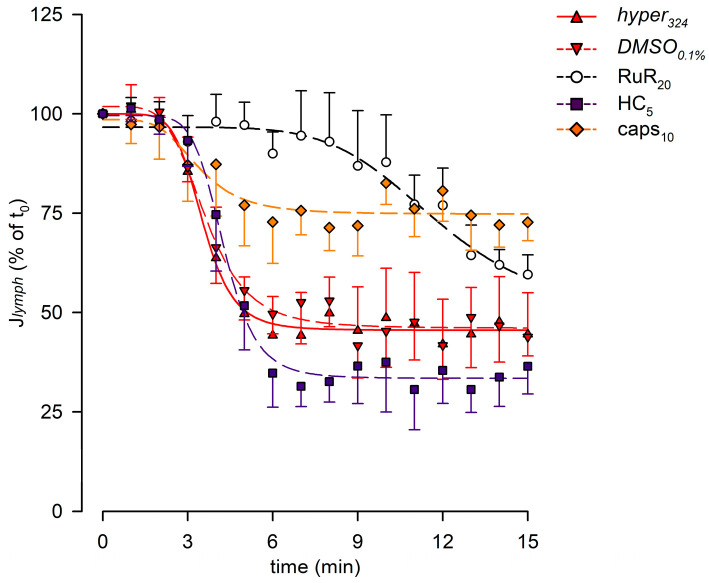
Plot of the overall effect of different channel blockers (RuR_20,_ hollow circles and black dashed line; HC_5,_ purple squares and purple dashed line; and caps_10,_ orange diamonds and orange dashed line) on *J_lymph_*, expressed as a percentage of *J_lymph_* at t_0_, compared to *hyper*_324_ (red up triangles and red solid line) or *DMSO*_0.1%_ (red down triangles and red dashed line). RuR_20_ significantly delayed the response to the hyperosmolar solution, while caps_10_ significantly attenuated the *J_lymph_* long-term reduction, although it did not affect the time of the response.

The analysis of end-diastolic diameters (Figure 6B), contraction amplitude (Figure 6C), and lymph flow rate (Figure 7) was performed for *hyper*_324_ and *DMSO*_0.1%_ groups and for lymphatic vessels exposed to the highest dose of any of the blockers tested (RuR_20_, HC_5_, and caps_10_). The overall analysis involved 36 lymphatic vessels having a mean d_D_ of 175.05 ± 9.66 μm (range 82.70–375.91 μm) at t_0_. Contracting lymphatics randomly assigned to different experimental groups were homogenously distributed, and no differences were found in mean d_D_ at t_0_ (168.15 ± 20.12 μm for *hyper*_324_, 170.58 ± 30.80 μm for *DMSO*_0.1%_, 177.34 ± 13.39 μm for RuR_20_, 178.70 ± 41.56 μm for HC_5_, and 180.88 ± 11.07 μm for caps_10_, *p* = 0.9923, one-way ANOVA). During the whole recording time, d_D_ was not significantly altered by the hyperosmolar environment alone or by the presence of any selective or nonselective channel blocker (*p* = 0.9826, one-way ANOVA).

The average contraction amplitude (Δd, Figure 6C) was 28.2 ± 2.0% of d_D_ at t_0_, displaying no changes related to osmolarity or due to drug application over time (*p* = 0.8849, one-way ANOVA).

The lymph flow rate (*J_lymph_*, Figure 7) was computed for each experimental group, displaying time-dependent decreases, which were all fitted with four-parameter sigmoidal curves (parameters are summarized in supplemental Table S3A). No differences were found in the presence of *DMSO*_0.1%_. Selective blocking of TRPV1 channels with caps_10_ reduced the long-term *J_lymph_* decrease, as *J_lymph_* at t_15_ was 72.7 ± 4.7%, a value significantly higher than *DMSO*_0.1%_ (*p* < 0.01, unpaired Student’s *t*-test, *n* = 14).

### 3.3. Effect of RuR, HC, and Capsazepine on Lymphatic Intrinsic Contractility in Hypotonic Solution

**Figure 8:**
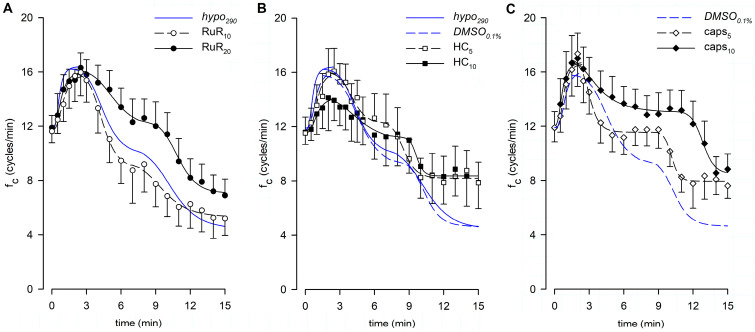
(**A**) Time course showing the effect of the nonselective TRPV1-6 channel blocker RuR in the 290-hypotonic solution on average f_c_ of spontaneously contracting lymphatics. No effect was found due to RuR_10_ (hollow circles and black dashed line), whereas RuR_20_ (filled circles and black solid line) delayed the response to the hypotonic environment. Time course of the effect of hypotonic solution alone (*hypo*_290_) is reported for reference (blue solid line). (**B**) Time course showing the effect of the TRPV4-selective blocker HC in the 290-hypotonic solution on average f_c_ of spontaneously contracting lymphatics. Early response in HC_5_ (hollow squares and black dashed line) was similar to *DMSO*_0.1%_ (blue dashed line), whereas exposure to HC_10_ (filled squares and black solid line) smoothed the shape of the early peak. (**C**) Time course showing the effect of the TRPV1-selective blocker caps in the 290-hypotonic solution on average f_c_ of spontaneously contracting lymphatics. No effects were found in the early increasing phase both in caps_5_ (hollow diamonds and black dashed line) and caps_10_ (filled diamonds and black solid line). At t_15,_ lymphatics challenged with caps_10_ displayed a f_c_ significantly higher than *DMSO*_0.1%_ (blue dashed line).

**Figure 9:**
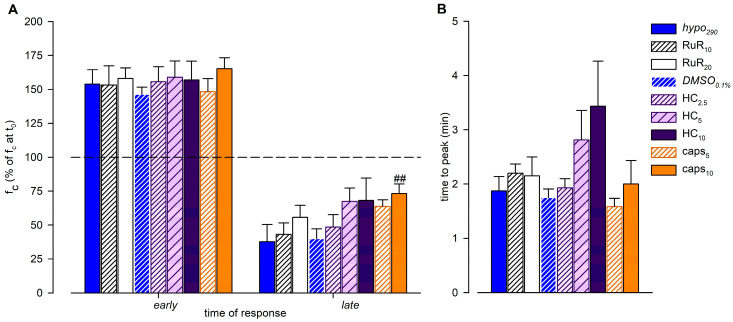
(**A**) Overall effect of different channel blockers in the 290-hypotonic solution, compared to *hypo*_290_ (blue solid bars) or *DMSO*_0.1%_ (blue striped bars), on early and late response of intrinsic f_c_ (expressed as a percentage of f_c_ displayed at t_0_). The effects of the nonselective TRPV1-6 channel blocker Ruthenium Red (10 μM white striped bars, 20 μM white solid bars), the selective TRPV4 channel blocker HC-067047 (2.5 μM purple narrow striped bars, 5 μM purple wide striped bars, and 10 µM purple solid bars), or the selective TRPV1 channel blocker capsazepine (5 μM orange striped bars, 10 μM orange solid bars) were tested. None of the channel blockers, at any concentration used, affected the early f_c_ peak (mean value of f_c_ peak for each tested vessel). caps_10_ induced a significant effect on the late plateau, with f_c_ being higher than *DMSO*_0.1%_ after 15 min of perfusion. ^##^ *p* < 0.01 vs. *DMSO*_0.1%_. For all tested conditions, early f_c_ peaks were significantly higher than 100%; late endpoints were significantly lower than 100% except caps_10_. (**B**) Effect of different channel blockers in the 290-hypotonic buffer on the time interval to peak (mean time required by each vessel to reach its own f_c_ peak). HC displayed a minor, but not significant, time delay in the response to hyposmolarity as the time to peak became longer.

Lymphatic vessels (*n* = 10, mean f_c_ at t_0_ = 11.6 ± 0.8 cycles/min) challenged with the nonselective TRPV1-6 channel blocker Ruthenium Red at a 10 μM dose (RuR_10_) displayed an early increase in intrinsic f_c_ to a peak of 17.7 ± 1.8 cycles/min (+53.2 ± 14.1% of t_0_,) followed by a decrease to a value of 5.2 ± 1.2 cycles/min within 15 min of total perfusion (Figure 8A), corresponding to a 56.9 ± 8.4% reduction of f_c_ at t_0_. Both mean f_c_ peak and late values were found to be significantly different from f_c_ at t_0_ (*p* < 0.01 vs. t_0_, paired Student’s *t*-test, *n* = 10, for both). A different set of spontaneously contracting vessels (*n* = 10, mean f_c_ at t_0_ = 11.9 ± 0.9 cycles/min) exposed to 20 μM RuR (RuR_20,_ Figure 8A) displayed a similar mean value of the early f_c_ peak of 18.3 ± 0.8 cycles/min (+58.1 ± 7.8%, Figure 9A, early) and a less pronounced time-dependent f_c_ decrease, attaining a slightly higher f_c_ of 6.9 ± 1.2 cycles/min at t_15_ (corresponding to a reduction of 44.3 ± 8.8% of f_c_ at t_0_, Figure 9A, late). However, even for RuR_20,_ both mean f_c_ peak and late values were significantly different from f_c_ at t_0_ (*p* < 0.01 vs. t_0_, paired Student’s *t*-test, *n* = 10, for both). Furthermore, no RuR-related differences were found in the mean early f_c_ peak or in the mean late f_c_ attained at t_15_ with respect to *hypo*_290_ (*p* = 0.9448 and *p* = 0.4234, respectively, one-way ANOVA). The early f_c_ increase for RuR_10_ and RuR_20_ data was fitted by sigmoidal curves (Figure 8A, parameters for data fitting are reported in supplemental Table S2A). The time interval to peak was similar for both data sets, being 2.20 ± 0.17 min for RuR_10_ and 2.15 ± 0.35 min for RuR_20_ (Figure 9B), displaying no differences with respect to *hypo*_290_ (*p* = 0.6902, one-way ANOVA).

The effect induced by the vehicle only on intrinsic f_c_ was tested also in the hypotonic environment, evaluating the response to 0.1% DMSO in 290 mOsm solution (*DMSO*_0.1%_, *n* = 6, mean f_c_ at t_0_ = 11.8 ± 1.7 cycles/min). Both the mean f_c_ peak and the late value were significantly different from the mean f_c_ displayed at t_0_ (*p* < 0.01, paired Student’s *t*-test, *n* = 6, for both). However, no DMSO-related effects were found with respect to *hypo*_290_ (Figure 8B). The mean value of the early f_c_ peak was 17.0 ± 1.9 cycles/min (+46.2 ± 5.4% of f_c_ at t_0_, Figure 9A, early; *p* = 0.5769 vs. *hypo*_290_, Student’s *t*-test, *n* = 14), and the time interval to peak was 1.75 ± 0.16 min (Figure 9B, *p* = 0.7194 vs. *hypo*_290_, Student’s *t*-test, *n* = 14). Intrinsic f_c_ attained the lower value of 4.9 ± 1.1 cycles/min at t_15_ (corresponding to a reduction of 60.0 ± 7.2% of f_c_ at t_0_, Figure 9A, late), as in *hypo*_290_ (*p* = 0.8838, unpaired Student’s *t* test, *n* = 14). Data fitting parameters for *DMSO*_0.1%_ experiments are reported in supplemental Table S2A,B.

A different set of lymphatic vessels (*n* = 7, mean f_c_ at t_0_ = 11.6 ± 1.0 cycles/min) challenged with the selective TRPV4 channel blocker HC-067047 at 2.5 μM (HC_2.5_) displayed an early f_c_ peak of 17.4 ± 0.6 cycles/min (*p* < 0.01 vs. t_0_, paired Student’s *t*-test, *n* = 7) corresponding to +55.6 ± 11.1% of f_c_ at t_0_ (Figure 9A, early, data points not shown). Then, f_c_ decreased to a lower value of 5.4 ± 1.0 cycles/min (*p* < 0.01 vs. t_0_, paired Student’s *t*-test, *n* = 7) corresponding to a 51.6 ± 9.2% decrease of f_c_ at t_0_ (Figure 9A, late), close to the value in *DMSO*_0.1%_. When lymphatics (*n* = 8, mean f_c_ at t_0_ = 11.5 ± 1.2 cycles/min) were exposed to 5 μM HC (HC_5_), the intrinsic f_c_ peak was 17.9 ± 1.6 cycles/min (*p* < 0.01 vs. t_0_, paired Student’s *t*-test, *n* = 8), corresponding to +58.9 ± 12.1% of f_c_ at t_0_ (Figure 8B and Figure 9A, early). Later, it reached a steady value of 7.9 ± 1.5 cycles/min (*p* < 0.05 vs. t_0_, paired Student’s *t*-test, *n* = 8) corresponding to a 32.5 ± 9.6% decrease of f_c_ at t_0_ (Figure 9A, late). The steady value was slightly higher but not significantly different from *DMSO*_0.1%_. Another group of lymphatics (*n* = 8, mean f_c_ at t_0_ = 11.6 ± 0.9 cycles/min) was exposed to an HC higher dose (10 μM, HC_10_), finding an early f_c_ peak of 18.1 ± 1.9 cycles/min (*p* < 0.01 vs. t_0_, paired Student’s *t*-test, *n* = 8), corresponding to + 57.0 ± 13.8% of f_c_ at t_0_ (Figure 8B and Figure 9A, early). The late f_c_ value was 7.9 ± 1.9 cycles/min (*p* = 0.082 vs. t_0_, paired Student’s *t*-test, *n* = 8), corresponding to a decrease of 31.7 ± 16.4% of f_c_ at t_0_ (Figure 9A, late). Overall, all of the tested HC doses did not significantly affect the intrinsic f_c_ early peak amplitude or the late f_c_ value with respect to *DMSO*_0.1%_ (*p* = 0.8916 and *p* = 0.2782, respectively, one-way ANOVA). On the other hand, HC induced a delay to reach the intrinsic f_c_ peak, which was found to be longer (2.81 ± 0.54 for HC_5_ and 3.44 ± 0.83 min for HC_10_), albeit not statistically different from *DMSO*_0.1%_ (Figure 9B, *p* = 0.1717, one-way ANOVA). Indeed, the time course of HC_10_ displayed an overall f_c_ peak smoother than both HC_2.5_ and HC_5_ (Figure 8B). Data fitting for early f_c_ rise displayed sigmoidal relationships for all HC data sets (parameters for data fitting are reported in supplemental Table S2A).

Spontaneously contracting lymphatics (*n* = 6, mean f_c_ at t_0_ = 11.9 ± 1.1 cycles/min) challenged with the selective TRPV1 channel blocker capsazepine 5 µM (caps_5_, Figure 8C) reached an early f_c_ peak of 17.8 ± 2.2 cycles/min (*p* < 0.01 vs. t_0_, paired Student’s *t*-test, *n* = 6) corresponding to +48.3 ± 9.7% of f_c_ at t_0_ (Figure 9A, early). Then, the late f_c_ value was 7.6 ± 0.9 cycles/min at t_15_ (*p* < 0.01 vs. t_0_, paired Student’s *t*-test, *n* = 6), equivalent to a reduction of 36.3 ± 5.0% of f_c_ at t_0_ (Figure 9A, late). Lymphatics (*n* = 9, mean f_c_ at t_0_ = 11.9 ± 1.2 cycles/min) exposed to capsazepine 10 μM (caps_10_, Figure 8C) displayed an f_c_ peak value of 19.1 ± 1.4 cycles/min (*p* < 0.01 vs. t_0_, paired Student’s *t*-test, *n* = 9) corresponding to +65.4 ± 7.9% of f_c_ at t_0_ (Figure 9A, early). After 15 min of perfusion, f_c_ reached a lower value of 8.8 ± 1.1 cycles/min (*p* < 0.01 vs. t_0_, paired Student’s *t*-test, *n* = 9), corresponding to a decrease of 26.8 ± 7.0% of f_c_ at t_0_ (Figure 9A, late), significantly less pronounced than *DMSO*_0.1%_ (*p* < 0.01, one-way ANOVA). In both cases, early f_c_ peaks and time intervals to peak (1.58 ± 0.15 min for caps_5_ and 2.00 ± 0.43 min for caps_10_, Figure 9B) were similar to *DMSO*_0.1%_ (*p* = 0.1842 and *p* = 0.6822, respectively, one-way ANOVA)_._ Overall, no differences were observed between caps_5_ and caps_10_ (Figure 8C).

**Figure 10:**
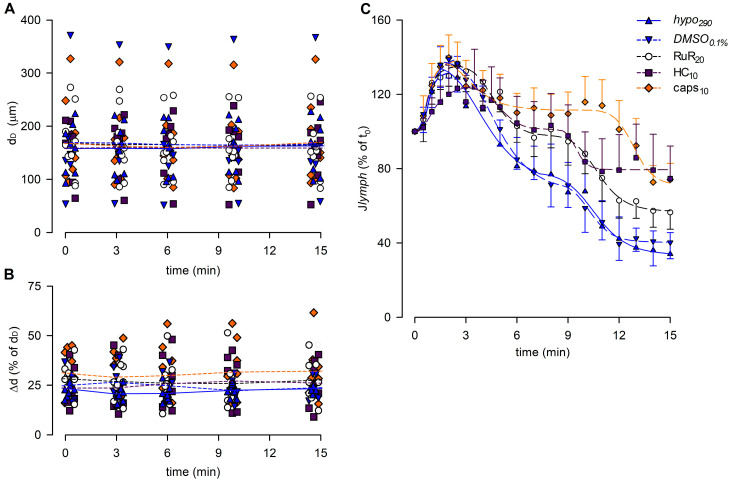
(**A**) Lymphatic vessels’ end-diastolic diameter and (**B**) contraction amplitude (Δd %) were not affected by the different drugs used (*hypo*_290,_ blue up triangles and blue solid lines; *DMSO*_0.1%,_ blue down triangles and blue dashed lines; RuR_20,_ hollow circles and black dashed lines; HC_10,_ purple squares and purple dashed lines; and caps_10,_ orange diamonds and orange dashed lines). (**C**) Plot of the overall effect of different channel blockers on *J_lymph_* (RuR_20,_ hollow circles and black dashed line; HC_10,_ purple squares and purple dashed line; and caps_10,_ orange diamonds and orange dashed line) expressed as a percentage of *J_lymph_* at t_0_, compared to *hypo*_290_ (blue up triangles and blue solid line) or *DMSO*_0.1%_ (blue down triangles and blue dashed line). caps_10_ significantly delayed the late response to the hypotonic environment, whereas HC_10_ significantly attenuated the *J_lymph_* long-term reduction.

The analysis of end-diastolic diameters (Figure 10A), contraction amplitude (Figure 10B), and lymph flow rate (Figure 10C) was performed for reference groups (*hypo*_290_ and *DMSO*_0.1%_) and for the highest dose of any drug tested (RuR_20_, HC_10_, and caps_10_). The overall analysis involved 41 spontaneously contracting diaphragmatic lymphatics whose mean d_D_ at t_0_ was 164.83 ± 10.43 μm (range 54.03–373.59 μm), similar to the mean d_D_ of vessels previously tested (*p* = 0.4785, Student’s *t*-test, *n* = 77). Mean d_D_ at t_0_ was comparable among different groups (158.69 ± 16.83 μm for *hypo*_290_, 169.84 ± 44.10 μm for *DMSO*_0.1%_, 168.22 ± 19.00 μm for RuR_20_, 158.82 ± 18.89 μm for HC_10,_ and 168.53 ± 26.54 μm for caps_10_, *p* = 0.9952, one-way ANOVA). Furthermore, no changes were induced by the hypotonic solution itself or by any of the different drugs used during the whole recording time period (*p* = 0.9983, one-way ANOVA).

The contraction amplitude was 26.5 ± 1.3% of d_D_ at t_0_ and was not affected by any of the conditions tested up to t_15_ (Δd, Figure 10B, *p* = 0.3325, one-way ANOVA).

Lymph flow (Figure 10C) displayed an early increase to a *J_lymph_* peak for all groups, fitted by four-parameter sigmoidal curves (parameters for data fitting are summarized in Table S3B). In all cases, the *J_lymph_* peak was followed by a time-dependent decrease described by four-parameter sigmoidal fitting (parameters for data fitting are summarized in Table S3C). *DMSO*_0.1%_ had no effect on the rising phase of *J_lymph_* as well as RuR_20_ and caps_10_. Conversely, the early rise for HC_10_ was slower and displayed a greater variability, resulting in a lower *J_lymph_* peak value of 124.0 ± 20.2%. *DMSO*_0.1%_ also did not affect the late decrease in *J_lymph_*, whereas the hypotonic-induced late reduction was less evident in the presence of RuR_20_ and HC_10_, albeit not significantly different with respect to reference traces. Only caps_10_ significantly reduced the late *J_lymph_* decrease (74.2 ± 8.7% vs. 40.3 ± 8.7% in *DMSO*_0.1%_, *p* < 0.05 vs. *DMSO*_0.1%_, Student’s *t*-test, *n* = 15).

### 3.4. Effect of DCPIB in the Hypotonic Environment on Lymphatic Intrinsic Contractility

**Figure 11:**
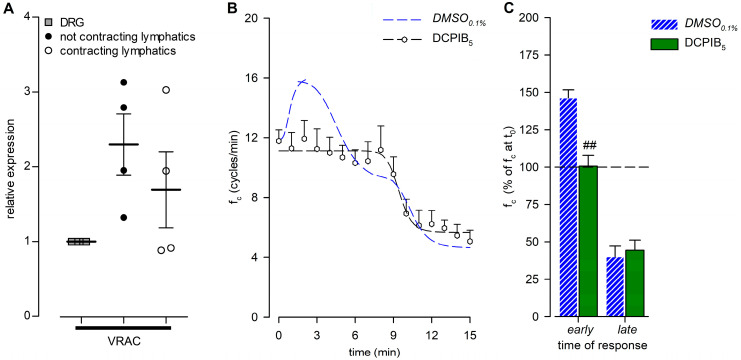
(**A**) Relative gene expression of VRACs in not-contracting (filled circles) and spontaneously contracting (hollow circles) diaphragmatic lymphatics, compared to the respective expression in dorsal root ganglions (DRGs; grey squares) used as positive controls. VRACs were similarly expressed in both contracting and not-contracting vessels; *n* = 4 animals for all genes tested. (**B**) Time course showing the effect of DCPIB (5 µM), the VRAC-selective inhibitor, in the 290-hypotonic solution on average f_c_ of spontaneous contracting lymphatics. DCPIB_5_ (hollow hexagons and black dashed line) completely abolished the early f_c_ response. *DMSO*_0.1%_ (blue dashed line) is reported for reference. (**C**) Early and late effects of DCPIB_5_ (green solid bars) on intrinsic f_c_ (expressed as a percentage of f_c_ displayed at t_0_) compared to *DMSO*_0.1%_ (blue striped bars), measured at the same interval as the *DMSO*_0.1%_ peak occurred. ^##^ *p* < 0.01 vs. *DMSO*_0.1%_.

Since selective and nonselective blocking of TRPV channels had no effect on f_c_ peak in lymphatics exposed to a hypotonic environment, we investigated a different kind of molecular sensor, such as VRACs. Their gene expression was tested by real-time PCR, which detected similar mRNA levels in both contracting and not-contracting diaphragmatic lymphatics (Figure 11A).

The functional analysis was performed on lymphatics (*n* = 8, mean f_c_ at t_0_ = 11.8 ± 0.7 cycles/min) challenged with the selective VRAC inhibitor DCPIB at a concentration of 5 µM (DCPIB_5_). DCPIB_5_ completely abolished the early f_c_ peak (Figure 11B), maintaining f_c_ stable for up to 7 min. Indeed, at the same time interval as the *DMSO*_0.1%_ peak occurred, the DCPIB_5_ mean f_c_ was 11.9 ± 1.2 cycles/min (*p* = 0.8691 vs. t_0_, paired Student’s *t*-test, *n* = 8), corresponding to +0.6 ± 7.2% of the starting f_c_, significantly lower than *DMSO*_0.1%_ (Figure 11C, early; *p* < 0.01, Student’s *t*-test, *n* = 14). After 15 min of DCPIB_5_ perfusion, f_c_ was 5.1 ± 0.8 cycles/min (*p* < 0.01 vs. t_0_, paired Student’s *t*-test, *n* = 8), displaying a 55.6 ± 6.7% reduction of the starting f_c_ (Figure 11C, late) similar to *DMSO*_0.1%_. DCPIB_5_ data fitting parameters are reported in supplemental Table S2B.

**Figure 12:**
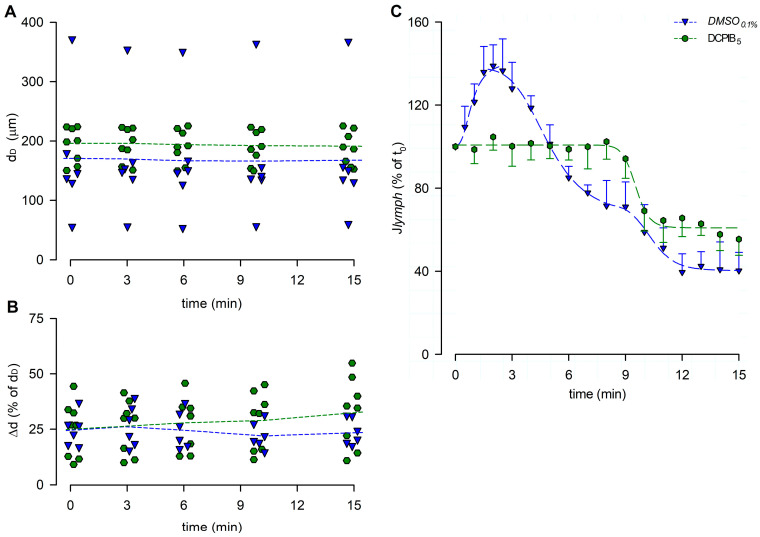
(**A**) Lymphatic vessels’ end-diastolic diameter and (**B**) contraction amplitude (Δd %) were not affected by DCPIB_5_ (green hexagons and green dashed lines). *DMSO*_0.1%_ (blue down triangles and blue dashed lines) is reported for reference. (**C**) Plot of the overall effect of DCPIB_5_ on *J_lymph_* (green hexagons and green dashed line) expressed as a percentage of *J_lymph_* at t_0_, compared to *DMSO*_0.1%_ (blue down triangles and blue dashed line). DCPIB_5_ completely abolished the early *J_lymph_* peak induced by the hypotonic environment.

The end-diastolic diameters analysis showed a mean d_D_ of 195.34 ± 10.81 μm at t_0_, not significantly different from d_D_ data obtained in all of the hypotonic groups previously described (*p* = 0.8876, one-way ANOVA). During the whole recording interval, no effects were induced by DCPIB_5_ on mean dD (Figure 12A, *p* = 0.9478 at t_15_, one-way ANOVA). Mean Δd was 25.12 ± 4.45% of d_D_ at t_0_, and it slightly increased to 32.97 ± 5.58% of d_D_ at t_15_ (Figure 12B), not significantly different from the hypotonic groups tested (*p* = 0.3381, one-way ANOVA).

The lymph flow rate computed for DCPIB_5_ (Figure 12C) lacked the early *J_lymph_* peak in response to the hypotonic solution. Conversely, a time-dependent late *J_lymph_* decrease was present, and it was fitted by a four-parameter sigmoidal curve (parameters for data fitting are summarized in supplemental Table S3C).

## 4. Discussion

### 4.1. Preliminary Findings on the Stability and Plasticity of Intrinsic Contractions

The intrinsic pumping mechanism of lymphatic vessels plays a variable role in the overall drainage effort of the lymphatic system, depending on the specific anatomical region. In body districts where the extrinsic mechanism is of minimal importance or absent, such as in soft tissues [48,49,50], the functional adaptability of the intrinsic mechanism becomes crucial for maintaining control over the hydraulic balance of the extracellular space. Apart from changes in extracellular fluid volume, alterations in its osmolarity can also impact this system. Our current study aims to clarify key characteristics of the short-term response of the intrinsic pumping mechanism to changes in osmolarity within the extracellular fluid.

Since lymph flow, from a purely mechanical standpoint, can be envisaged as the result of stroke volume ejected in one single contraction multiplied by the contraction frequency of the lymphangion itself, the steadiness of contraction frequency, and stroke volume in undisturbed diaphragmatic lymphatics, our experimental results demonstrate that intrinsic pumping exhibits a consistent contraction rate over a 25 min period (Figure 2). Therefore, any significant deviation from this stable behaviour can confidently be attributed to the specific experimental conditions employed in subsequent tests, and any variation in *J_lymph_* thus underlies a physiological response whose significance is to be assessed.

Furthermore, the investigation into the response of the intrinsic mechanism to fluid osmolarity reveals a monotonic sigmoidal decrease in f_c_ in response to hyperosmolarity, while a biphasic pattern with an initial f_c_ increase followed by a later decrease characterizes the response to hyposmolarity. This time-dependent response is consistent across experiments conducted at both 35 °C and 37 °C, suggesting that while osmolarity and temperature can influence the contraction rate of the lymphatic muscle [29,31,51], their mechanisms of action are independent of each other. This underscores the functional adaptability of lymph drainage supported by the intrinsic mechanism.

### 4.2. Response to a Hyperosmolar Environment and Putative Involved Receptors

TRPV channels are known to be activated by osmolarity changes, either directly or through the resulting cell volume variation within the initial minutes of exposure to a different osmotic environment [51,52,53,54]. While our experiments did not allow us to discriminate between these two possibilities, using the nonselective TRPV1-6 antagonist RuR, we observed an inhibition of the response to hyperosmolarity in terms of contraction frequency. Considering that TRPV1 and TRPV4 are primarily expressed in the walls of both contracting and not-contracting lymphatic vessels, as evident from Figure 5, the application of the specific TRPV4 blocker HC (panel B) had no effect on the normal response, but the application of capsazepine, a selective TRPV1 blocker (panel C), was able to abolish the decrease in f_c_. Moreover, the analysis of end-diastolic and systolic vessel diameters revealed no significant differences between the various blocker applications (Figure 6B,C). Consequently, this suggests that the role played by TRPV1 is primarily confined to the pacemaker mechanism, rather than exerting a broader effect on LM tone, either tonically (as d_D_ remains unchanged) or phasically (as Δd remains unchanged).

In analogy with cardiac function, the lymph flow rate can be derived as the product of contraction frequency and stroke volume, which was computed in the lymphangion using a well-established method developed by our research group [29]. Given the interplay between f_c_ and stroke volume, which is dependent on the Δd during each contraction, data presented in Figure 7 demonstrate that the selective TRPV1 blockage exerted by capsazepine does not alter the time course of the overall response but significantly affects the steady-state flow. In principle, one would expect similar behaviour in both conditions since RuR has a broader blocking effect compared to capsazepine.

### 4.3. Response to a Hyposmolar Environment and Putative Involved Receptors

The response of lymphatic vessels to a hyposmolar environment is more complex compared to the hyperosmolar case, being characterized by an early increase in f_c_ (Figure 3B) followed by a gradual but continuous decrease to a steady value towards the end of the 15 min recording period (Figure 3B).

**Figure 13:**
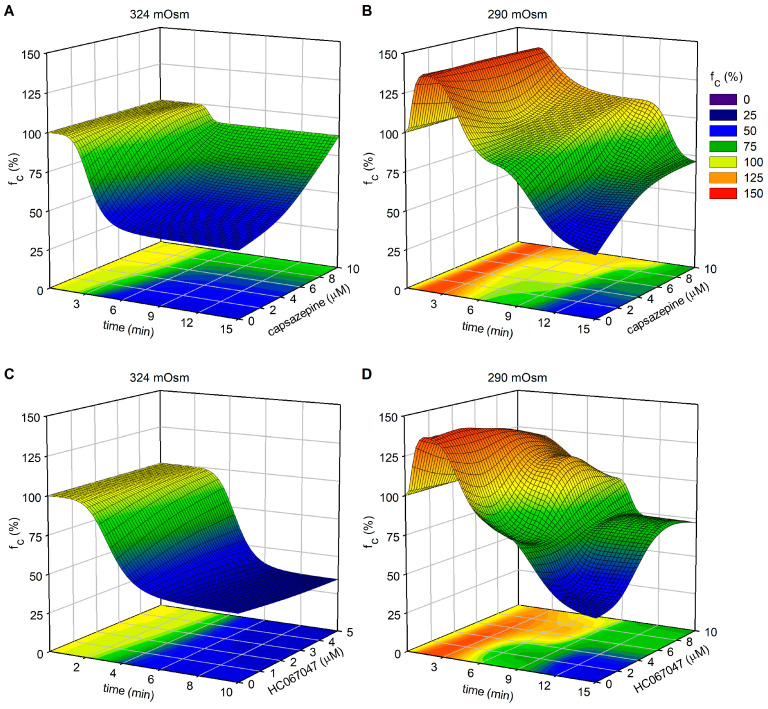
Plot of intrinsic f_c_ modifications (z-axis—expressed as a percentage of f_c_ at t_0_) as a function of elapsed time (x-axis) and TRPV1 (capsazepine, panels (**A**,**B**)) or TRPV4 (HC-067047, panels (**C**,**D**)) channel blockers’ concentration (y-axis), combined with different fluid osmolarities (324 mOsm for panels (**A**,**C**) and 290 mOsm for panels (**B**,**D**)).

Nonspecific TRPV blockage by RuR did not result in any significant difference either in the early or late responses compared to *hypo*_290_ (Figure 8A) in terms of f_c_. In addition, none of the TRPV blockers used were able to eliminate the early f_c_ response to the hyposmolar environment (Figure 9A and Figure 13B,D), ruling out the putative role of TRPV4 and TRPV1 in the induction of this early increase in contraction frequency. However, the blockage of TRPV1 by capsazepine, at the higher tested dose, significantly prevented the decrease in f_c_ that characterizes the late response (Figure 9A).

Moreover, no statistically significant differences were found in d_D_ values or Δd (Figure 10A,B) when vessels were exposed to the hyposmolar solution alone or in the presence of various TRPV blockers. This suggests that the mechanism altering intrinsic f_c_ does not involve the generation of force from LM, either tonically or phasically. The computed lymph flow rate demonstrated that blockages of TRPVs by RuR, HC, and capsazepine influenced the overall time-dependent variation in *J_lymph_*. In all three conditions, the late response exhibited a less pronounced decrease in *J_lymph_* compared to their respective reference group (Figure 10C).

To further investigate the mechanism underlying the early f_c_ increase, a different putative involved receptor has been considered. Indeed, both contracting and non-contracting lymphatic vessels express VRAC channels (Figure 11A), a potential sensor of the cell swelling induced by hypotonic conditions [55]. The application of DCPIB, a selective VRAC inhibitor, completely blocked the early response alone, with no effect on the late response to the hyposmolar environment (Figure 11B,C). An analysis of vessel diameters showed that even DCPIB did not affect d_D_ or Δd (Figure 12A,B) and that *J_lymph_* was mainly affected during the early response (Figure 12C), while the late phase was similar to the reference condition.

## 5. Conclusions

Lymph formation and propulsion are essential mechanisms that contribute to maintaining fluid homeostasis in interstitial spaces and serosal cavities. The extent of fluid drainage by the lymphatic system must be balanced with fluid filtration from blood capillaries to achieve a steady state. The lymph flow rate due to the intrinsic pumping mechanism alone, which relies on the spontaneous contractions of lymphatic muscle in collecting lymphatic vessels, can be modelled as the product of the actual contraction frequency and stroke volume. Therefore, any stimulus that alters either or both of these parameters has the potential to affect the extent of fluid drainage. The osmolarity of the surrounding environment plays a role in this balance, as an increase or decrease in fluid osmolarity can result from diminished or increased fluid filtration, among other possibilities.

This study has shown that the response of collecting lymphatic vessels, in terms of contraction frequency and the lymph flow rate, is influenced by modifications in the microenvironment osmolarity. By applying selective and nonselective blockers of TRPV and VRAC channels, the key molecular sensors involved in the responses to hyperosmotic and hyposmotic stimuli were identified. While the long-term responses for both conditions involve a reduction in contraction frequency and lymph flow rate, the hyposmotic environment induces an early increase in contraction frequency and lymph flow. In particular, this early response may be the result of an increase in fluid filtration unaccompanied by a proportional increase in the amount of solutes, leading to the accumulation of hyposmotic interstitial fluid. On the other hand, an increase in interstitial osmolarity may reflect a reduced body fluid volume, and, therefore, no increase in fluid removal would be expected unlike, in contrast, a reduction in lymphatic drainage for the maintenance of correct fluid homeostasis. The late reduction in contraction frequency and the lymph flow rate remains a puzzling question, especially in the case of the hyposmotic environment, where a sustained increase in lymph formation is expected.

Both hypertonic and hypotonic conditions elicit a decrease in lymph flow, primarily driven by a reduction in contraction frequency. This shared behaviour suggests a potential purpose of limiting the transport of lymph with altered osmolarity within the lymphatic vascular tree and, subsequently, into the bloodstream in the case the local interstitial fluid environment fails to return to normal within a few minutes following the initial perturbation. However, the implications of this particular response remain speculative. The transient VRAC-mediated increase in contraction frequency and lymph flow under hypotonic conditions gives rise to an unresolved question within this framework. Nevertheless, its temporal duration is so limited compared to the sustained decline in lymph flow during the steady state that its true significance in terms of the overall osmolarity of the transported lymph remains a matter of controversy.

In a previous study [29], we investigated the different and peculiar responses of diaphragmatic and dermal lymphatics to fluctuations in local tissue temperature. Therefore, the responses observed in diaphragmatic lymphatics may not be quantitatively and/or qualitatively analogous in other body districts. Furthermore, the impact of hyperosmolarity and/or hyposmolarity alone on the efficacy of various blocking agents can introduce an additional variable. Indeed, in another paper [30], we documented a TRPV4-mediated complete blockade of the temperature effect under isosmotic conditions using lower concentrations of HC and RuR than those employed in the present investigation. However, the experimental model used in this study did not allow for longer-term observation, and further investigations are needed to understand any subsequent adjustments in the response. From a cellular and molecular perspective, it is fascinating to observe how lymphatic vessels express different classes of ionic channels, enabling them to sense a broad range of osmolarity values around the normal set point. This ability allows them to modulate intrinsic contraction frequency without altering vessel diameter, thus preserving the mean hydraulic resistance to lymph flow.

## Figures and Tables

**Table 1:** Solutions used in ex vivo experiments.

Solution Name	D-glucose (mM)	D-mannitol (mM)	Drugs	Measured Osmolarity (mOsm)
**A. preliminary experiments**				
*storage*	33	none	none	308
*control*_308_	16.5	16.5	none	308
**B. HYPERosmolar solutions**				
*control*_308_	16.5	16.5	none	308
*hyper*_324_	16.5	33	none	324
RuR			Ruthenium Red	
RuR_10_	16.5	33	10 µM	324
RuR_20_	16.5	33	20 µM	324
HC			HC-067047	
HC_2.5_	16.5	33	2.5 µM	324
HC_5_	16.5	33	5 µM	324
*DMSO*_0.1%_	16.5	33	Dimethyl sulfoxide 0.1%	324
caps			capsazepine	
caps_5_	16.5	33	5 µM	324
caps_10_	16.5	33	10 µM	324
**C. HYPO-osmolar solutions**				
*control*_308_	16.5	16.5	none	308
*hypo*_290_	16.5	1	none	290
RuR			Ruthenium Red	
RuR_10_	16.5	1	10 µM	290
RuR_20_	16.5	1	20 µM	290
HC			HC-067047	
HC_2.5_	16.5	1	2.5 µM	290
HC_5_	16.5	1	5 µM	290
HC_10_	16.5	1	10 µM	290
*DMSO*_0.1%_	16.5	1	Dimethyl sulfoxide 0.1%	290
caps			capsazepine	
caps_5_	16.5	1	5 µM	290
caps_10_	16.5	1	10 µM	290
DCPIB_5_	16.5	1	DCPIB 5 µM	290

**Table 2:** Oligonucleotides used in Real-Time PCR assay.

Oligo Name	Sequence (5′–3′)
β-actin	
forward	GACAGGATGCAGAAGGAGATTACTG
reverse	CTCAGGAGGAGCAATGATCTTGAT
VRACs	
forward	GGCCACCCTCTTCAAGATCC
reverse	ATGTCGCTGTAGCTGCTCTC

## Data Availability

All original data are available on request from andrea.moriondo@uninsubria.it.

## Supplementary Materials

The following supporting information can be downloaded at: https://www.mdpi.com/article/10.3390/biology12071039/s1, Supplemental Video S1: Representative video recording of a lymphatic vessel bathed with the isotonic and hyperosmolar solutions; Supplemental Video S2: Representative video recording of a lymphatic vessel bathed with the isotonic and hyposmolar solutions; Supplemental Figure S1: Representative tracings of the diameter profile over time of lymphatic vessels exposed to the different conditions summarized in Figure 6; Supplemental Figure S2: Representative tracings of the diameter profile over time of lymphatic vessels exposed to the different conditions summarized in Figure 10 and Figure 12; Supplemental Table S1: Parameters for f_c_ data fitting, hyperosmolar experimental groups; Supplemental Table S2: Parameters for f_c_ data fitting, hyposmolar experimental groups; Supplemental Table S3: Parameters for *J_lymph_* (%) data fitting.
