# Supplementary material for: Fluid Osmolarity Modulates the Rate of Spontaneous Contraction of Lymphatic Vessels and Lymph Flow by Means of a Cooperation between TRPV and VRAC Channels
Source: Biology (Basel). 2023 Jul 23;12(7):1039. doi: 10.3390/biology12071039 (PMC10376700; doi:10.3390/biology12071039)
Supplement: Supplementary file 1 [file biology-12-01039-s001.zip › biology-2452691-supplementary.pdf]

Supplements

Contents:

- Supplemental Video S1: Representative video recording of a lymphatic vessel bathed with the isotonic and hyperosmolar solutions.
- Supplemental Video S2: Representative video recording of a lymphatic vessel bathed with the isotonic and hyposmolar solutions.
- Supplemental Figure S1: Representative tracings of the diameter profile over time of lymphatic vessels exposed to the different conditions summarized in Figure 6.
- Supplemental Figure S2: Representative tracings of the diameter profile over time of lymphatic vessels exposed to the different conditions summarized in Figure 10 and Figure 12.
- Supplemental Table S1: Parameters for f_c data fitting, hyperosmolar experimental groups.
- Supplemental Table S2: Parameters for f_c data fitting, hyposmolar experimental groups.
- Supplemental Table S3: Parameters for J_{lymph} (%) data fitting.

Video S1: Representative video recording of a lymphatic vessel bathed with the isotonic and hyperosmolar solutions.

The panel on the left (308 mOsm) shows the contractile behaviour of an FITC-filled lymphatic tract video recorded in an isotonic condition. The panel on the right (324 mOsm) shows the behaviour of the very same vessel after 15 minutes of perfusion with $hyper_{324}$. Scale bar is 150 µm.

Video S2: Representative video recording of a lymphatic vessel bathed with the isotonic and hyposmolar solutions.

The panel on the left (308 mOsm) shows the contractile behaviour of an FITC-filled lymphatic tract video recorded in an isotonic condition. The panel in the middle (290 mOsm E) shows the behaviour of the very same vessel at the intrinsic f_c peak (~ 2 minutes of perfusion with $hypo_{290}$ - *early*). The panel on the right (290 mOsm L) shows the behaviour of the very same vessel after 15 minutes of perfusion with $hypo_{290}$ (*late*). Scale bar is 150 µm.

Figure S1

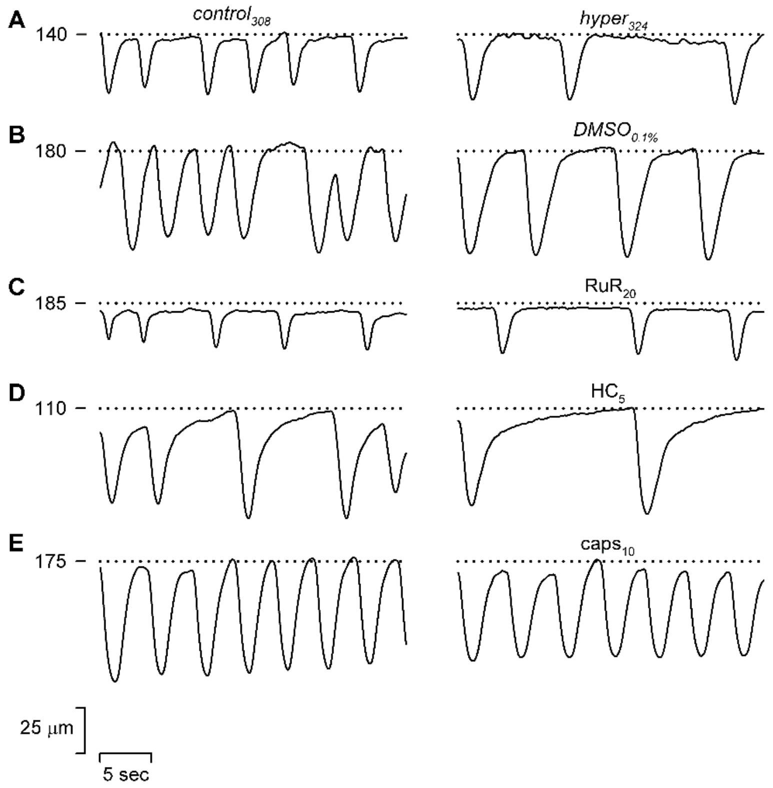

Representative tracings of the diameter profile over time of lymphatic vessels exposed to the different conditions summarized in Figure 6.

The panels on the left show the contractile behaviour of different lymphatic vessels obtained under isosmotic *control*$_{308}$ conditions. The panels on the right show the behaviour of the very same lymphatics after 15 minutes of perfusion in the different tested conditions for hyperosmolar experimental groups. **A** *hyper*$_{324}$, **B** *DMSO*$_{0.1\%}$, **C** RuR$_{20}$, **D** HC$_{5}$, and **E** caps$_{10}$.

Figure S2

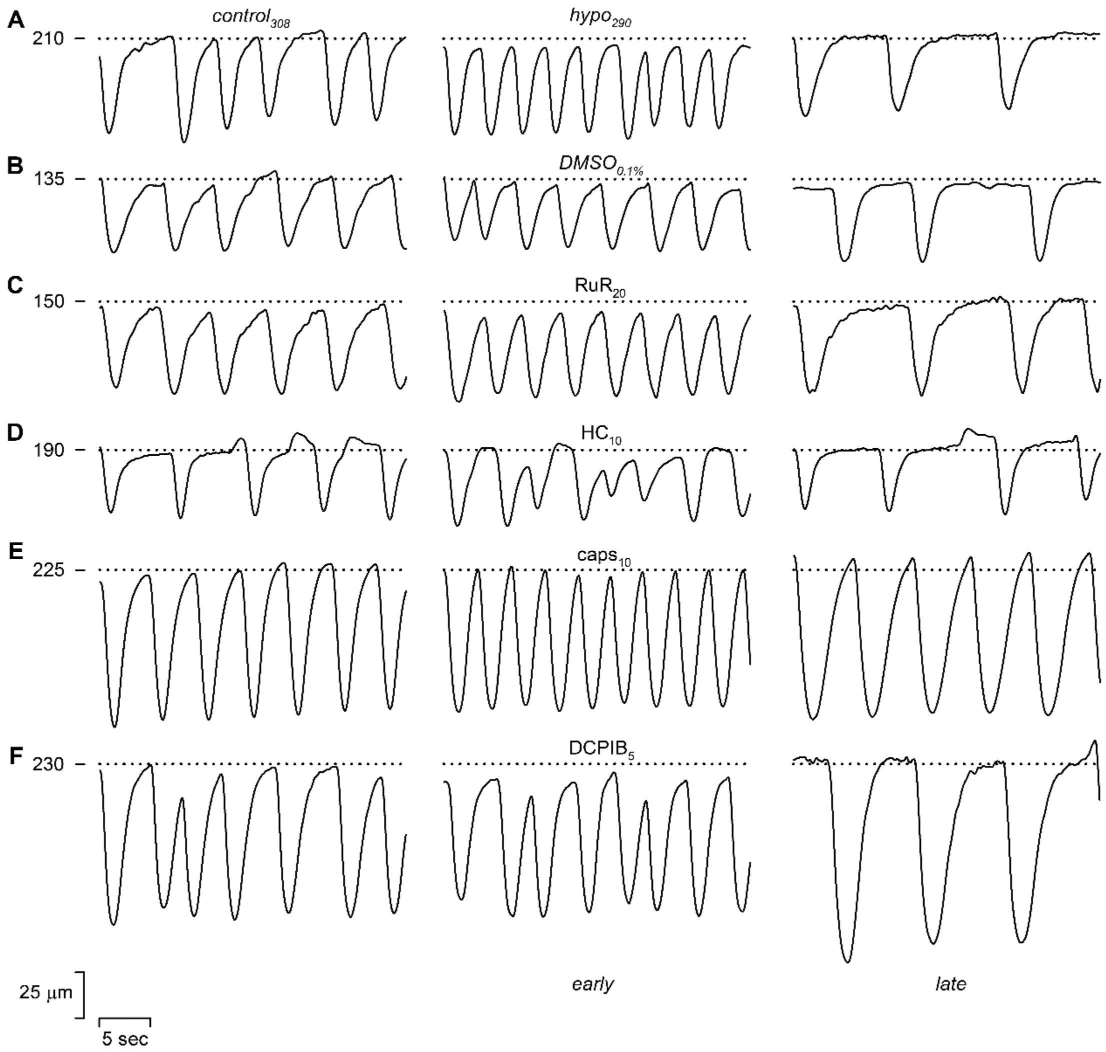

Representative tracings of the diameter profile over time of lymphatic vessels exposed to the different conditions summarized in Figure 10 and Figure 12.

The panels on the left show the contractile behaviour of different lymphatic vessels obtained under isosmotic *control*$_{308}$ conditions. The panels in the middle show the behaviour of the very same vessels at the time interval of intrinsic f_c peak (*early*) in the different tested conditions for hyposmolar experimental groups. The panels on the right show the behaviour of the very same lymphatics after 15 minutes of perfusion (*late*). **A** *hypo*$_{290}$, **B** *DMSO*$_{0.1\%}$, **C** RuR$_{20}$, **D** HC$_{10}$, **E** caps$_{10}$, and **F** DCPIB$_{5}$.

Table S1 Parameters for f_c data fitting, hyperosmolarity experimental groups.

**Parameters for sigmoidal fit of f_c decrease to endpoint**					
**Experimental group**	**top** (cycles/min)	**bottom** (cycles/min)	**f_{c50}** (min)	**HS**	**r^2**
$hyper_{324}$	11.2 ± 0.2	4.7 ± 0.1	3.39 ± 0.12	5.3 ± 0.8	0.99
RuR_{10}	11.6 ± 0.3	4.2 ± 0.2	4.08 ± 0.18	3.5 ± 0.5	0.99
RuR_{20}	11.3 ± 0.1	6.9 ± 0.5	10.04 ± 0.66	4.6 ± 0.9	0.98
$DMSO_{0.1\%}$	11.0 ± 0.2	5.0 ± 0.1	3.63 ± 0.14	5.0 ± 0.9	0.98
$HC_{2.5}$	11.2 ± 0.2	4.2 ± 0.1	4.15 ± 0.11	6.0 ± 0.9	0.99
HC_5	11.6 ± 0.1	4.0 ± 0.1	4.37 ± 0.07	8.2 ± 0.9	0.99
$caps_5$	11.6 ± 0.3	6.5 ± 0.2	4.72 ± 0.27	7.2 ± 2.5	0.95

Table S2 Parameters for f_c data fitting, hyposmolarity experimental groups.

A. Parameters for sigmoidal fit of f_c rise to early peak

**Experimental group**	**top** (cycles/min)	**bottom** (cycles/min)	**f_{c50}** (min)	**HS**	**r^2**
$hypo_{290}$	16.4 ± 0.3	11.8 ± 0.2	0.63 ± 0.08	-3.4 ± 1.2	0.98
RuR_{10}	15.9 ± 0.5	11.7 ± 0.3	1.00 ± 0.14	-2.9 ± 1.1	0.98
RuR_{20}	16.2 ± 0.3	11.9 ± 0.3	0.81 ± 0.11	-2.7 ± 0.8	0.99
$DMSO_{0.1\%}$	16.1 ± 0.5	11.9 ± 0.3	0.92 ± 0.13	-3.3 ± 1.4	0.98
$HC_{2.5}$	16.8 ± 0.2	11.6 ± 0.1	1.03 ± 0.03	-2.7 ± 0.2	0.99
HC_{5}	16.0 ± 0.4	11.7 ± 0.2	1.05 ± 0.11	-3.7 ± 1.4	0.98
HC_{10}	14.2 ± 0.4	11.6 ± 0.2	1.02 ± 0.17	-2.9 ± 1.4	0.98
$caps_{5}$	16.6 ± 0.6	11.9 ± 0.5	0.79 ± 0.16	-3.6 ± 2.0	0.96
$caps_{10}$	16.8 ± 0.5	12.1 ± 0.3	0.65 ± 0.10	-2.9 ± 1.2	0.98

B. Parameters for sigmoidal fit of f_c decrease to endpoint

**Experimental group**	**top** (cycles/min)	**bottom** (cycles/min)	**$f_{c50,1}$** (min)	**HS_1**	**$f_{c50,2}$** (min)	**HS_2**	**r^2**
$hypo_{290}$	16.2 ± 0.2	4.4 ± 0.1	4.51 ± 0.30	5.7 ± 1.9	10.39 ± 0.10	9.0 ± 0.6	0.97, 0.99
RuR_{10}	15.9 ± 0.1	5.3 ± 0.3	4.39 ± 0.11	6.8 ± 1.0	9.57 ± 0.35	8.5 ± 2.3	0.99, 0.97
RuR_{20}	16.2 ± 0.1	7.0 ± 0.2	5.42 ± 0.39	5.2 ± 1.6	10.91 ± 0.15	13.2 ± 2.1	0.98, 0.99
$DMSO_{0.1\%}$	15.8 ± 0.2	4.6 ± 0.2	4.79 ± 0.26	5.1 ± 1.2	10.36 ± 0.17	14.9 ± 3.1	0.98, 0.99
$HC_{2.5}$	16.3 ± 0.1	5.4 ± 0.2	4.24 ± 0.11	5.6 ± 0.7	10.30 ± 0.18	26.1 ± 9.8	0.99, 0.97
HC_{5}	16.0 ± 0.2	8.2 ± 0.2	4.14 ± 0.28	6.7 ± 2.7	8.53 ± 0.16	15.5 ± 3.7	0.94, 0.98
HC_{10}	14.2 ± 0.1	8.4 ± 0.2	4.72 ± 0.46	3.9 ± 1.2	9.62 ± 0.18	30.5 ± 11.7	0.96, 0.97
$caps_{5}$	16.7 ± 0.3	8.0 ± 0.2	3.07 ± 0.16	7.6 ± 2.7	10.15 ± 0.13	31.6 ± 14.2	0.97, 0.98
$caps_{10}$	17.1 ± 0.2	8.5 ± 0.4	3.63 ± 0.23	3.5 ± 0.7	12.85 ± 0.19	25.2 ± 8.1	0.98, 0.98
	**top** (cycles/min)	**bottom** (cycles/min)			**f_{c50}** (min)	**HS**	**r^2**
$DCPIB_{5}$	11.1 ± 0.2	5.7 ± 0.3			9.45 ± 0.19	18.7 ± 5.9	0.97

Table S3 Parameters for J_{lymph} (%) data fitting.

A. Hyperosmolar environment—Parameters for sigmoidal fits of J_{lymph} (%) decrease to endpoint

Experimental group	top J (%)	bottom J (%)	J_{50} (min)	HS	r^2
$hyper_{324}$	99.9 ± 1.4	45.6 ± 0.8	3.53 ± 0.09	6.6 ± 0.9	0.99
RuR_{20}	96.6 ± 1.1	49.7 ± 10.3	11.77 ± 1.04	6.0 ± 1.7	0.97
$DMSO_{0.1\%}$	101.8 ± 2.1	46.1 ± 1.2	3.64 ± 0.15	4.9 ± 0.8	0.98
HC_5	99.5 ± 1.6	33.5 ± 1.0	4.28 ± 0.09	7.5 ± 1.0	0.99
$caps_{10}$	98.6 ± 2.7	74.8 ± 1.5	3.38 ± 0.44	4.4 ± 2.2	0.88

B. Hyposmolar environment—Parameters for sigmoidal fits of J_{lymph} (%) early rise to peak

Experimental group	top J (%)	bottom J (%)	J_{50} (min)	HS	r^2
$hypo_{290}$	132.2 ± 3.0	100.3 ± 2.5	0.83 ± 0.10	-4.0 ± 1.6	0.99
RuR_{20}	136.1 ± 4.0	99.7 ± 3.6	0.78 ± 0.13	-3.1 ± 1.3	0.99
$DMSO_{0.1\%}$	141.5 ± 5.9	100.6 ± 3.5	0.89 ± 0.17	-2.6 ± 1.1	0.98
HC_{10}	126.0 ± 1.3	99.8 ± 0.8	1.20 ± 0.07	-2.5 ± 0.4	0.99
$caps_{10}$	139.2 ± 3.5	100.3 ± 3.0	0.81 ± 0.11	-3.0 ± 1.0	0.99

C. Hyposmolar environment—Parameters for sigmoidal fits of J_{lymph} (%) decrease to endpoint

Experimental group	top J (%)	bottom J (%)	$J_{50,1}$ (min)	HS_1	$J_{50,2}$ (min)	HS_2	r^2
$hypo_{290}$	135.2 ± 1.9	33.4 ± 3.0	4.33 ± 0.34	3.7 ± 0.8	10.61 ± 0.26	11.4 ± 2.7	0.99, 0.98
RuR_{20}	135.7 ± 1.3	56.9 ± 1.8	4.74 ± 0.21	6.9 ± 1.9	10.76 ± 0.15	14.0 ± 2.4	0.99, 0.99
$DMSO_{0.1\%}$	137.7 ± 1.0	40.3 ± 1.6	4.91 ± 0.14	4.7 ± 0.5	10.32 ± 0.18	16.3 ± 4.1	0.99, 0.98
HC_{10}	124.0 ± 0.8	79.4 ± 1.7	5.08 ± 0.19	8.2 ± 2.2	9.48 ± 0.28	29.0 ± 15.6	0.98, 0.93
$caps_{10}$	138.3 ± 1.9	70.8 ± 6.0	3.46 ± 0.36	4.5 ± 1.8	12.88 ± 0.32	22.3 ± 10.2	0.92, 0.96

Experimental group	top J (%)	bottom J (%)			J_{50} (min)	HS	r^2
$DCPIB_5$	100.7 ± 1.0	60.9 ± 1.4			9.54 ± 0.13	26.9 ± 6.5	0.98
